# Fire performance and spalling mitigation in high-strength and eps-modified mortars under transient thermal exposure

**DOI:** 10.1038/s41598-025-17637-3

**Published:** 2025-09-12

**Authors:** A. Y. F. Ali, Sabry A. Ahmed, Passant Youssef, A. M. Abdel-Wahab, M. S. El-Feky

**Affiliations:** 1 Materials Engineering Department, Zagazig University, Zagazig, 44519 Egypt; 2 Civil Engineering Program, German University in Cairo, Cairo, Egypt; 3 Department of Civil Engineering, National Research Centre, Cairo, Egypt

**Keywords:** High-strength cement mortar, Expanded polystyrene (EPS), Short-term elevated temperature exposure, Thermal spalling, Thermal insulation, Engineering, Materials science

## Abstract

High-strength cementitious composites are highly susceptible to explosive spalling at elevated temperatures, necessitating innovative fire resilience strategies. This study investigates the dual role of expanded polystyrene (EPS) beads as both lightweight aggregate and spalling mitigator in mortars subjected to short-term, rapid thermal exposure. High-strength mortar (HSM) and EPS-modified mortar were exposed to temperatures up to 600 °C with brief dwell times (10–30 min), followed by furnace or water cooling. Residual properties and microstructural evolution were systematically evaluated. Results revealed a fundamental temperature-dependent shift in EPS performance. At 400 °C, EPS incorporation prevented explosive spalling across all cooling regimes by generating porosity that dissipated internal vapor pressure, while plain HSM suffered severe spalling. Both mixtures showed strength gains at 200 °C due to thermal activation, though EPS mortar exhibited attenuated improvements. At 400 °C, EPS mortar experienced progressive strength loss attributed to bead decomposition and resulting porosity increases. EPS-enhanced thermal insulation was confirmed through significantly reduced core temperatures. However, under extreme conditions (600 °C), EPS decomposition triggered catastrophic spalling. Microstructural analysis showed EPS-induced voids transitioned from beneficial pores to critical defects with increasing temperature, while elemental analysis confirmed degradation was primarily physical rather than chemical. These findings demonstrate EPS’s viability for spalling mitigation in moderate fire scenarios, though its application requires careful temperature management. The study underscores the importance of evaluating fire mitigation materials under realistic transient heating conditions.

## Introduction

Preserving concrete buildings against damage from fire is critical since it will significantly reduce losses. Thus, it is critical to concentrate research efforts on fire safety. Ordinary Portland cement (OPC)-based concrete is the most extensively used material in the building sector^1,2^. Concrete has intrinsic fire resistance due to its poor thermal conductivity and noncombustibility. Elevated temperatures can degrade structural integrity, including load-carrying capability, durability, and fire resistance. The primary sources of these problems include high-temperature vapor pressure, thermal stresses, chemical changes in the cement paste, and discrepancies in thermal behavior between the matrix and coarse particles^3^. High-strength cement composites (HSCC) are more susceptible to strength and durability deterioration than normal-strength concretes (NSC). Additionally, it is more susceptible to explosive spalling^3–5^. The explosion of HSCC in a fire poses a major risk to human life and the integrity of the structure. One way to describe the nature of this explosion is the abrupt and severe rupture of a heated HSCC surface layer. This is because the presence of free and bonded water, which evaporates as the temperature rises, prevents the HSCC from releasing the accumulated internal pressure. The primary reason for this is the HSCC’s (extremely dense microstructure) poor permeability^3,6^.

Lightweight concrete (LWC) offers various benefits over normal-weight concrete. These include a greater strength-to-weight ratio, lower earthquake danger, improved fire resistance, and increased thermal insulation^7–11^. LWC reduces energy needs for HVAC systems, accounting for up to 60% of total building energy use, due to its exceptional thermal insulation characteristics^12,13^. Based on the ACI 318 − 14^14^, LWC with compressive strength exceeding 17 MPa and densities ranging from 1120 to 1920 kg/m^3^ can be used for structural applications. Though LWC has improved fire resistance, as previously stated, the risk of explosion persists at elevated temperatures, particularly when it is produced based on a high-strength base material to compensate for the strength reduction caused by light aggregate^15^. Nonetheless, literature that studies the potential for explosion spalling of LWC based on a high-strength matrix is rare. For instance, Aslani and Ma^15^ reported that, after exposure to high temperatures using a heating rate of 5 °C/min and soaking time at the target temperature for 1 h, the high-strength lightweight self-compacting concrete (HSLWSCC), which was made of scoria as a light aggregate and had a density of 2208 kg/m3, exhibited major explosions that occurred at 300 °C. Furthermore, the peak compressive strength of HSLWSCCs was attained at a temperature of 300 °C, and then the strength decreased^15^. To mitigate the potential explosion hazards of high-strength concrete, various polymeric fibers, including polypropylene^16^, polyvinyl alcohol^17^, acrylic^18^, nylon^19^, and recycled polyethylene terephthalate (PET)^18^, were added to the concrete as a means of releasing pressure. The purpose of incorporating these fibers into concrete is to utilize their ability to melt as the temperature increases. This melting process creates more voids inside the concrete, which helps to relieve internal pore pressure caused by the presence of free water and chemically bonded water, which evaporates as the temperature rises.

To the best of our knowledge, no study has investigated the ability of EPS to mitigate the explosion spalling of lightweight, high-strength-based concrete. EPS is a type of thermoplastic polymer that has a closed cellular structure. EPS has remarkable characteristics, including its low density, thermal insulating characteristics, hydrophobic nature, and resistance to chemical degradation when subjected to acids and alkalis^20,21^. Adding EPS beads to concrete mixtures offers a realistic solution for conserving natural aggregate resources and mitigating the environmental consequences of EPS trash accumulation^22^.

Several research studies have investigated the effect of elevated temperature due to air cooling on the residual mechanical properties of HSCC^3,15,23–40^. Matesov et al.^24^ found that the compressive strength and modulus of elasticity of HSCM increased at temperatures between 200 and 300 °C, then gradually decreased till reaching 600 °C. Furthermore, following exposure to a temperature of 400 °C, the compressive strength of HSCC was reduced by roughly 40% compared to its strength at room temperature^28–30,39^. Nevertheless, Castillo and Durrani^25^ demonstrated a remarkable enhancement in the residual compressive strength of HSCC at a temperature of 400 °C. Previous studies^41–51^ investigated the influence of water cooling on the mechanical characteristics of HSCC. At temperatures above 200 °C, water cooling has been shown to have a higher residual compressive strength than air cooling because it compacts the microstructure and makes it less porous by filling fissures and cavities with rehydrated compounds, thereby improving residual strength^41,44–50^. In contrast, water cooling appears to have a lower residual compressive strength than air cooling. This was ascribed to heat shock and volumetric increase caused by rehydrated compounds, which form new fractures and reduce the remaining compressive strength^42,43,51^. Unfortunately, the literature investigating the effect of water and air cooling on the mechanical properties and the potential for explosion of LWC produced by incorporating EPS in high-strength baes martial is missing.

### Research significance

During a fire, concrete structural elements may undergo short periods of intense heating, especially with the development of fire extinguishing systems. Previous literature didn’t investigate how short heating periods affect the mechanical behavior of HSCC, as they all used soaking times ranging from 1 h to 3 h. The current study aims to fill this gap in previous literature by exploring the effect of water cooling and furnace cooling after short soaking times of 10, 20, and 30 min at target temperatures of 200, 400, and 600 °C, with a relatively high heating rate of 10 °C/min, on the mechanical properties and the potential for explosion of HSCM and LWHSC produced by incorporating EPS in the HSCM. The fundamental mechanisms by which EPS beads either mitigated or did not mitigate the spalling are revealed. Furthermore, the effect of using EPS on enhancing thermal properties is investigated.

## Experimental work

### Experimental program

Two mixtures, HSCM and LWHSC, were examined to assess their performance under exposure to elevated temperatures. The specimens were exposed to 200, 400, and 600 °C for varying durations (10, 20, and 30 min) and subsequently cooled using either water or furnace methods. Mechanical properties, including compressive strength, splitting tensile strength, and impact resistance, were measured at room temperature after 7 and 28 days of curing, as well as following post-fire exposure at 28 days. Each test condition was replicated across three identical specimens to ensure statistical reliability. The experimental program implemented in this study aligns with the methodology outlined in the reference^11^, with the key variation involving the utilization of two distinct mortar mixtures.

The LWHSC mixture was prepared by replacing 50% of the sand volume in the HSCM mixture with an equivalent volume of EPS. The exact proportions of constituent materials required to produce 1 m³ of both HSCM and LWHSC mixtures are detailed in Table 1. A standardized notation system was adopted for clarity: C0 designates the HSCM mixture, whereas C50 identifies the EPS-modified LWHSC mixture. Soaking durations (10, 20, 30 min) are represented numerically, while F and W indicate furnace and water-cooling methods, respectively. For instance, C50/30/W signifies an LWHSC sample exposed thermally for 30 min, then water-quenched.

**Table 1:** Constituent material quantities for HSCM and LWHSC mixtures (kg/m³).

Mix Code	Cement	Silica fume	Sand	EPS	Water	Superplasticizer
HSCM	710	70	1354	-	203	23.4
LWHSC	710	70	677	3.346	203	23.4

### Materials characterization

**Fig.1:**
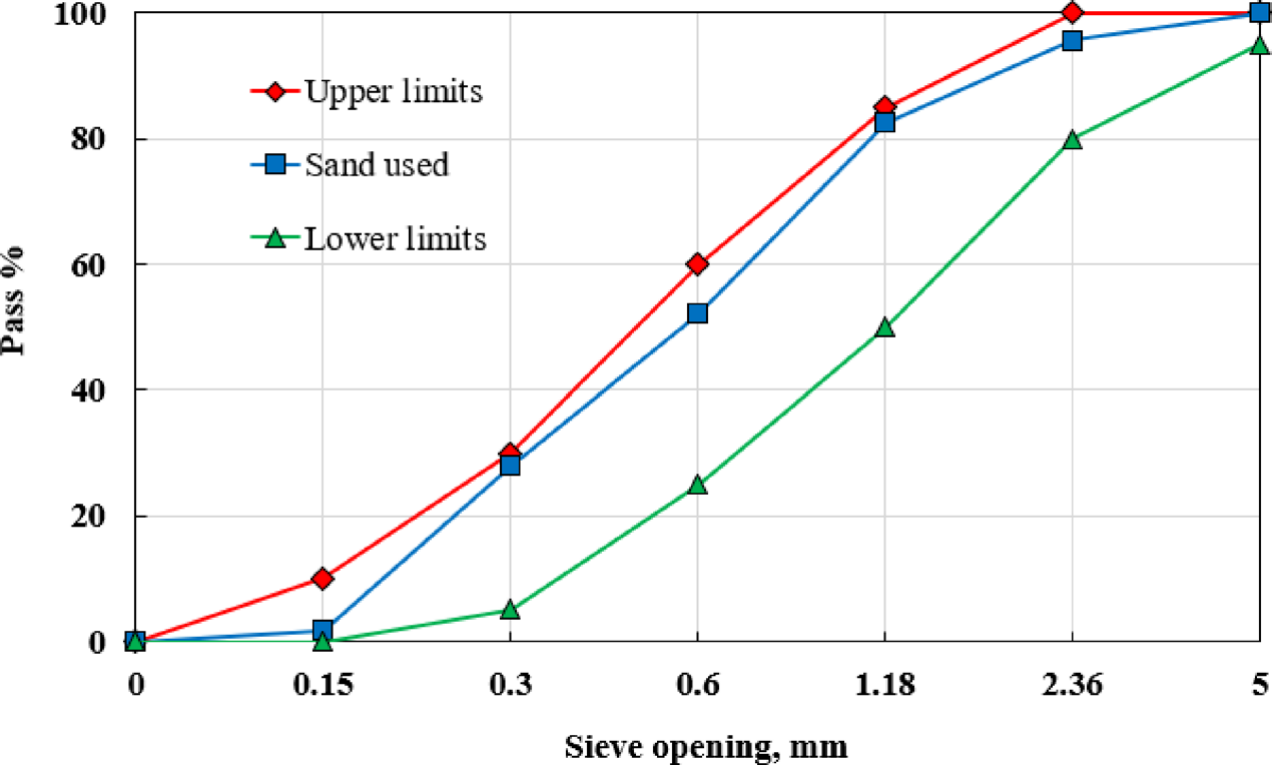
Grading curve of sand following Egyptian standard specifications^52^.

Ordinary Portland Cement (CEM I 52.5 N) served as the binding material in the preparation of both HSCM and LWHSC mixtures. Silica fume (SF), characterized by a particle size of 7 μm, was incorporated as a mineral additive. The physical properties of the cement and silica fume, as specified by the manufacturer, are summarized in Table 2.. A superplasticizer (Sikament-NN) was introduced to both mixtures at a dosage equivalent to 3% of the cement weight. Natural, washed sand with a particle size distribution between 0.15 mm and 5 mm was utilized as the fine aggregate in both mixes, exhibiting a specific gravity of 2.65 and a fineness modulus of 2.25. The grading curve of the fine aggregate is illustrated in Fig. 1. In the LWHSC mixture, EPS beads, locally sourced and ranging from 3 mm to 5 mm in diameter with a unit weight of 13.1 kg/m³, were employed as the lightweight aggregate.

**Table 2:** Chemical composition of OPC and SF as provided by the manufacturer.

Materials Chemical properties									
	SiO_2_	AL_2_O_3_	Fe_2_O_3_	CaO	MgO	SO_3_	K_2_O	Na_2_O	L.O. I
OPC	21.2	4.67	5.05	64.73	1.5	2.05	0.22	0.3	2.6
SF	88.9	0.29	0.94	0.2	0.36	5.12	–	0.52	3.4

### Mixing procedures

The mixing procedure for HSCM followed a defined sequence. First, predetermined quantities of cement, silica fume, and sand were dry-mixed in a mechanical pan mixer for a duration of 2 min, without the inclusion of water. Following this initial step, water and superplasticizer were gradually incorporated, and mixing continued for approximately 5 min. For the LWHSC, the same procedure was applied with an additional step: EPS beads were incorporated into the mix 3 min after the water was added, followed by two more minutes of mixing. Once homogeneous mixing was achieved, the fresh mixtures were cast into cubic molds. In this study, the lightweight concrete compositions were formulated to maintain adequate paste volume, ensuring sufficient viscosity to prevent EPS bead segregation^15,53^. The spatial distribution of EPS within the specimen cross-section is illustrated in Fig. 3. Specimens were demolded 24 h after casting and subsequently cured in water at 20 ± 3 °C for a period of 27 days.

**Fig.2:**
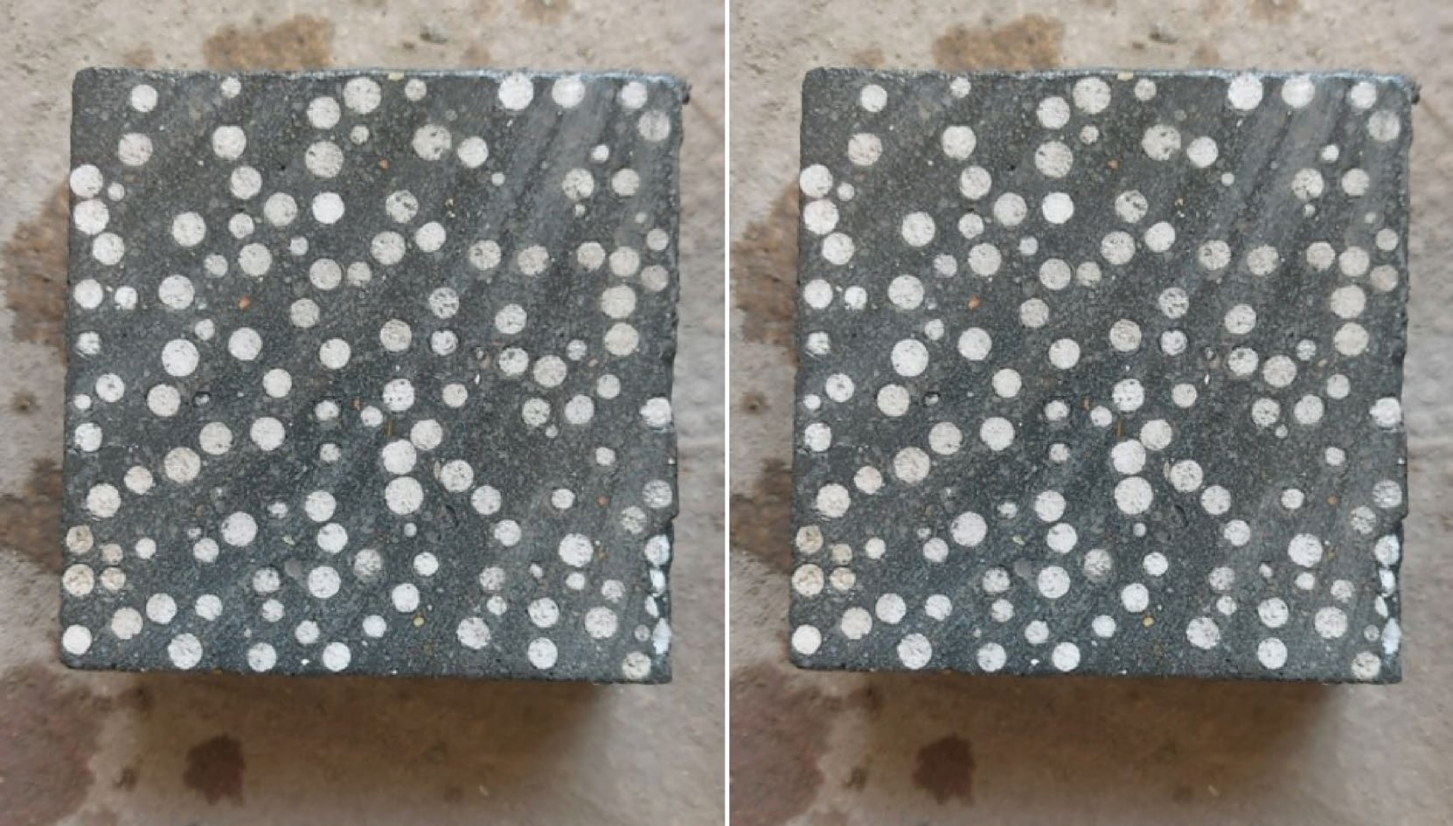
Cross-sectional distribution of EPS beads within the tested specimens.

### Heating and cooling procedures

**Fig.3:**
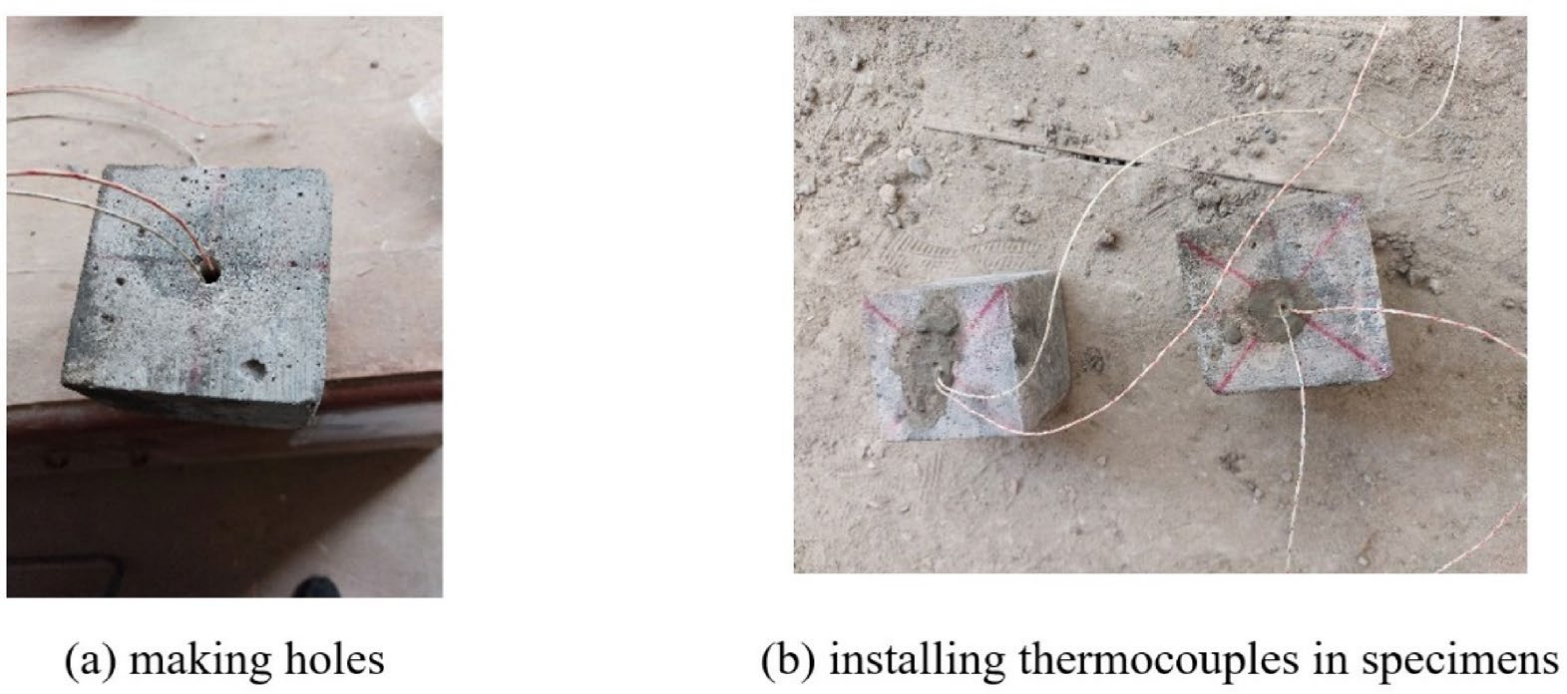
The procedures for installing type K thermocouples into cubes.

After completing 27 days of water curing, the specimens were exposed to laboratory air for one day to allow surface drying. Prior to thermal exposure, all specimens were oven-dried at 105 °C for two hours to mitigate the risk of explosive spalling. Heating was then conducted using a compact electric furnace equipped with vertically aligned heating coils on both sides of the chamber. The furnace chamber was capable of accommodating two cube specimens, each measuring 100 × 100 × 100 mm. The applied heating rate was 10 °C/min, which, according to references^41,42^, is considered relatively high for specimens of this scale due to the resulting pronounced thermal gradients. The applied heating rate, which is higher than standardized ISO regimes, was selected for two primary reasons: firstly, to replicate the rapid temperature rise observed in transient real-fire scenarios (e.g., compartment flashes, electrical cabinet fires); and secondly, to align with methodologies used in previous studies^32,41^ that intentionally employ higher heating rates to exacerbate internal pore pressure build-up, thereby allowing for a more severe evaluation of the efficacy of spalling mitigation strategies like EPS incorporation. Once the target temperature was reached, the specimens were held at that temperature for predetermined durations of 10, 20, or 30 min. Subsequently, two distinct cooling regimes were applied. In the furnace cooling method, specimens were left to cool gradually within the semi-open furnace until they reached ambient conditions. In the water cooling procedure, the hot specimens were promptly removed from the furnace and immersed in room-temperature water for five minutes, then left to continue cooling in air until equilibrium was reached.To track the internal temperature during heating and to evaluate the insulating effect of EPS, a type K thermocouple was embedded at the center of a designated sacrificial cube in each test group. The thermocouple was connected to a data logger that recorded the core temperature at five-second intervals. Due to the limited size of the heating chamber, the furnace temperature closely matched the surface temperature of the specimens.The thermocouple installation process involved drilling a hole at the center of the cube after seven days of water curing, with a depth of 50 mm and a diameter of 8 mm. The thermocouple was inserted into this cavity, which was then sealed using fresh mortar of the same composition. The specimens were subsequently returned to water curing for another 27 days prior to thermal testing, as illustrated in Fig. 3. The procedures employed for specimen heating, cooling, and thermocouple installation aligned with the methodology described in reference^11^.

### Mechanical and microstructural characterization

Following thermal exposure at elevated temperatures with varying soaking durations, and subsequent cooling using either the furnace or water immersion method, all specimens were allowed to stabilize under ambient conditions for 24 h before conducting the residual strength tests. The residual compressive strength was assessed in accordance with BS EN 12390-3^54^, using cube specimens of dimensions 100 × 100 × 100 mm. Residual splitting tensile strength was evaluated following the procedures outlined in BS EN 12390-6^55^. Impact resistance was determined using the drop weight method described in ACI 544.2R-89^56^, wherein a 4.5 kg steel ball was repeatedly dropped from a height of 450 mm under gravity (9.81 m/s²). For each specimen, the number of blows required to initiate visible surface cracking (N_i_) and to cause complete structural failure (N_f_) was recorded. The first was identified by the appearance of a fine, observable hairline crack on the top surface, while the latter corresponded to the complete separation or displacement of specimen parts from the body. The values of N_i_ and N_f_ were considered as indicators of initial and final crack resistance, respectively. The experimental protocol for residual strength assessments and SEM/EDS analysis closely followed those employed in the reference^11^. As a control, the compressive strength, splitting tensile strength, and impact resistance of unexposed specimens were measured at 7 and 28 days under ambient conditions. For microstructural analysis, scanning electron microscopy (SEM) was carried out at the National Research Centre in Egypt using a JEOL JSM-6510LV microscope with a maximum magnification of 300,000×. A range of magnifications (1000×, 5000×, 10,000×, and 15,000×) was employed to investigate surface morphology. SEM samples were extracted from crushed cube specimens 10 mm beneath the exposed surface following the 28-day compressive strength test. These fragments were dried at 70 °C until constant mass was achieved, then mounted on sample holders using carbon adhesive. To enhance surface imaging resolution, the samples were gold-coated using a sputter coating evaporator. In addition, elemental composition was assessed through energy-dispersive X-ray spectroscopy (EDS) utilizing the Oxford X-Max 20 system.

## Results and discussion

### Potential for explosion spalling

**Fig.4:**
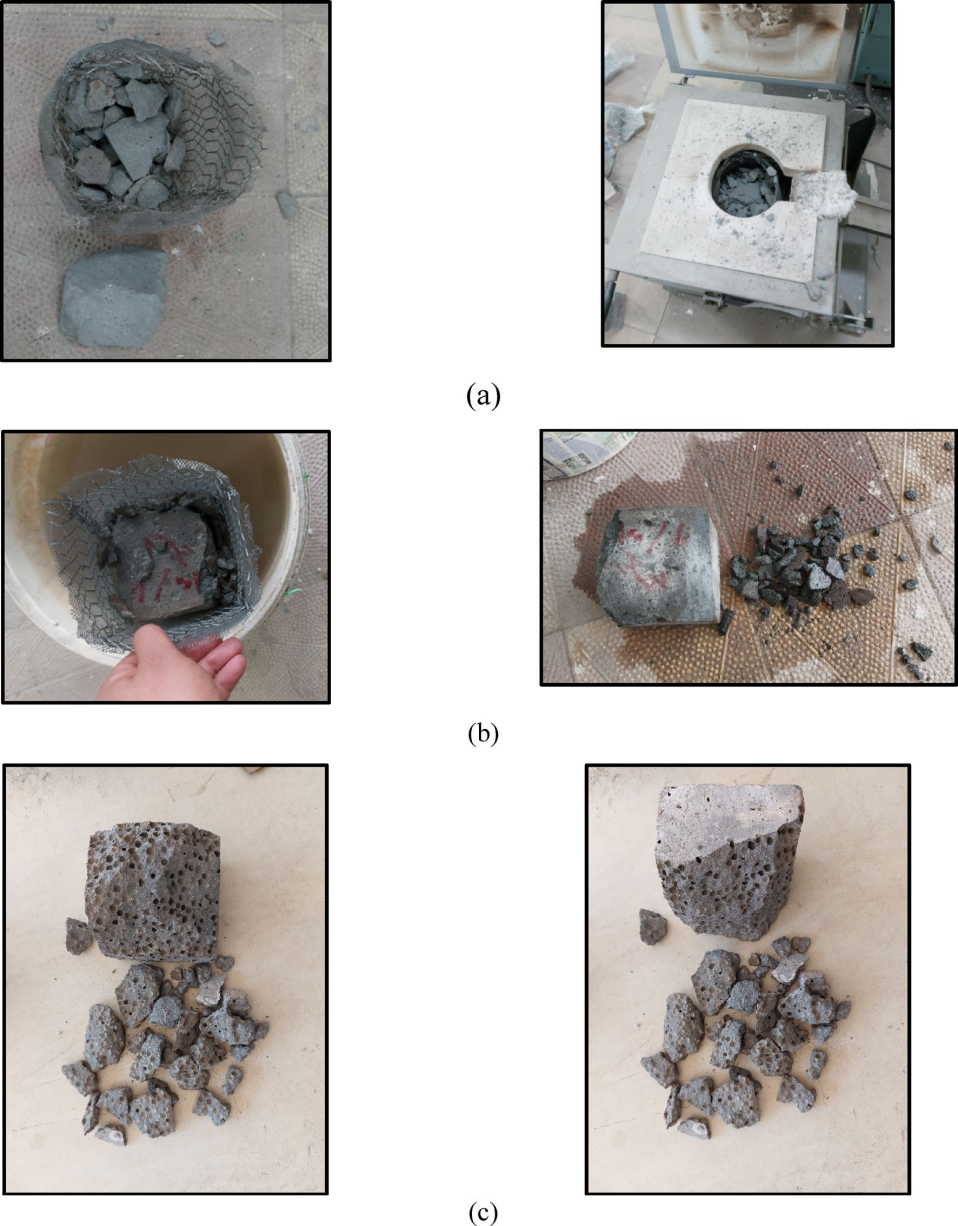
(**a**) explosion of C0 at 400 °C at a heating period of 30 min in the oven; (**b**) explosion of C0 at 400 °C at a heating period of 10 min when cooled in water; and (**c**) explosion of C50 at 550 °C during heating before reaching 600 °C.

This section addresses the influence of elevated temperatures and cooling methods (furnace and water cooling) at 200, 400, and 600 °C, alongside varying soaking durations of 10, 20, and 30 min, on the risk of explosive spalling in C0 and C50 specimens. Explosive spalling refers to the sudden and forceful detachment of surface layers from heated concrete when subjected to high thermal stresses^15^. The findings revealed that C0 specimens withstood explosive spalling up to 400 °C for a heating duration of 30 min under furnace cooling. Nevertheless, when exposed to 400 °C for 27–30 min (surface = 400 °C, core ≈ 219 °C), approximately 10% of the furnace-heated specimens exhibited explosive spalling during heating, as depicted in Fig. 4a. Spalling led to fragmentation of the specimens into pieces measuring 2–6.5 cm in length and 1.5–5 cm in thickness, with only about 35% of the original volume preserved. This behavior is primarily attributed to the dense microstructure, which hinders the dissipation of internal pressure generated by the evaporation of both physically and chemically bound water. Moreover, a substantial thermal gradient of 181.3 °C between the surface and the core introduced significant thermal stresses. The combination of internal vapor pressure and these stresses triggered explosive failure in the specimens. In the case of water cooling, no spalling was observed in C0 specimens at 200 °C across all soaking durations. However, all specimens heated to 400 °C for 10, 20, or 30 min (surface = 400 °C, core = 148.75 °C for the 10-minute case) and subsequently quenched in water experienced quench-induced explosive spalling during cooling. The fragmentation resulting from the spalling event produced debris measuring 1–3.5 cm in length and 0.5–2.5 cm in thickness, leaving approximately 70% of the original specimen volume intact, as illustrated in Fig. 4b. This phenomenon is likely due to thermal shock induced by the sudden cooling, which generated additional thermal stresses surpassing the mortar’s tensile strength, in conjunction with vapor pressure buildup. Consequently, results under water cooling at 400 °C could not be obtained. Similarly, results at 600 °C were not recorded since all specimens spalled explosively during heating before reaching the target temperature.

Unlike the C0 mix, the incorporation of EPS in the C50 specimens effectively suppressed explosive spalling at 400 °C across all heating durations, regardless of the cooling method applied. This behavior is attributed to the decomposition of EPS beads, which introduced additional porosity and microcracks within the high-strength matrix, enhancing its ability to dissipate internal pressure. However, at 600 °C, all C50 specimens spalled violently around 550 °C, i.e., explosive spalling during heating (~ 55 min of heating, core temperature could not be measured due to the violent nature of the event) during furnace heating. The spalling event resulted in fragmentation into debris measuring 1–4 cm in length and 0.8–3.5 cm in thickness, leaving approximately 65% of the original specimen volume intact, as illustrated in Fig. 4c. This can be explained by the combined effect of rapid heating, the matrix’s low permeability, and the EPS content, which is composed of approximately 95% air by volume, all of which contributed to elevated internal vapor pressure^57^. The resulting thermal stresses ultimately exceeded the tensile capacity of the matrix, leading to explosive failure.

### Influence of soaking duration and cooling method on the compressive strength of the C0 mixture

The influence of elevated temperatures (200, 400, and 600 °C), combined with soaking durations of 10, 20, and 30 min at each temperature level, and two distinct cooling methods (furnace and water cooling), on the residual compressive strength of C0 mix cube specimens was assessed. The average compressive strength values obtained before heating and after exposure to the various thermal conditions are presented in Table 1.. At ambient conditions, the compressive strength reached 61 MPa and 76 MPa at 7 and 28 days, respectively.

**Fig.5:**
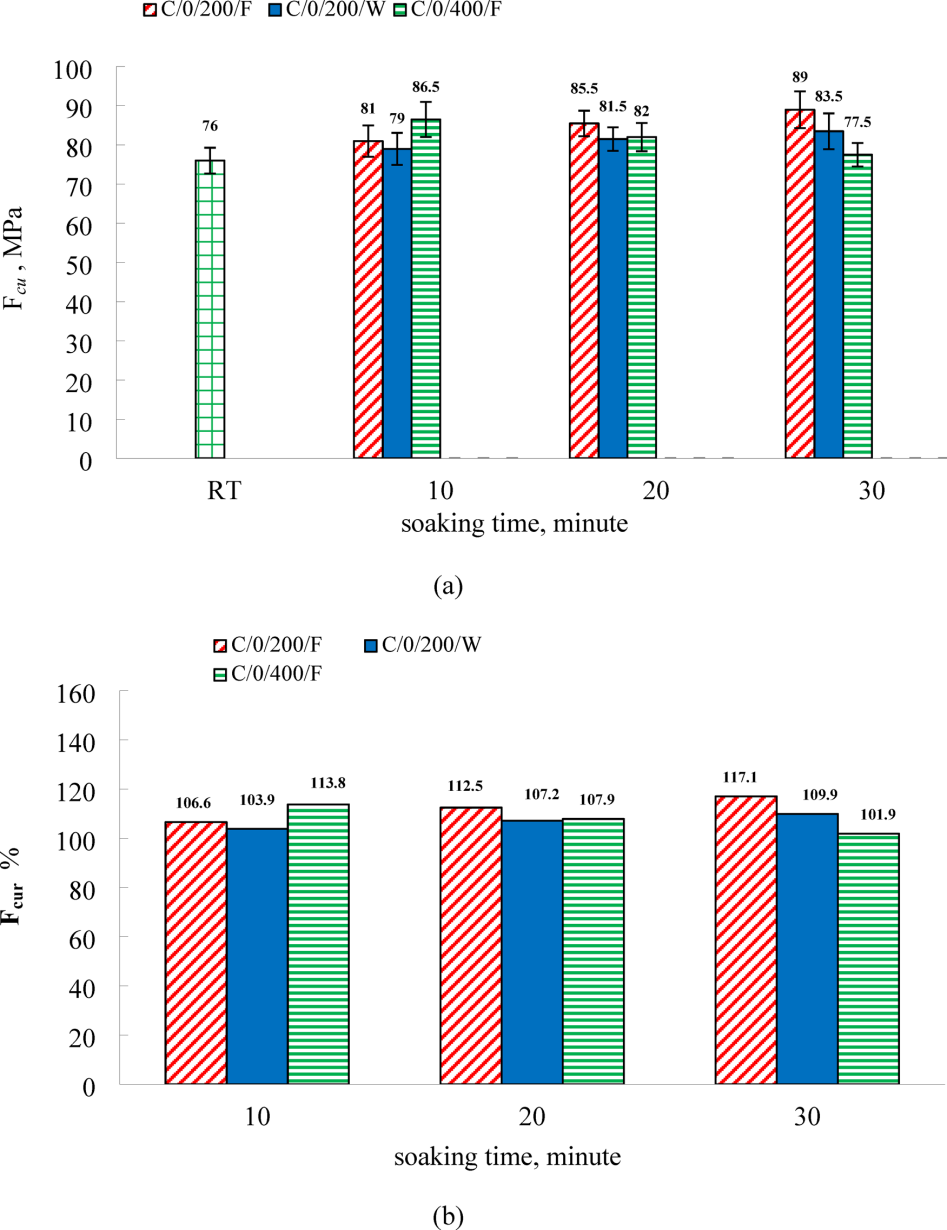
(**a**) The residual compressive strength for C0, (**b**) relative residual compressive strength for C0.

As illustrated in Fig. 5, exposure to 200 °C led to an increase in compressive strength relative to room temperature values. For furnace-cooled specimens, the strength improved by 6.6%, 12.5%, and 17.1% at soaking durations of 10, 20, and 30 min, respectively. Water-cooled specimens showed corresponding increases of 3.9%, 7.2%, and 9.9%. These results indicate that prolonged exposure time enhances residual compressive strength under both cooling regimes, with furnace cooling producing more pronounced gains than water cooling. Similar observations at 200 °C were reported by Kara and Arslan^47^. The observed strength improvement at this temperature is likely due to continued hydration of previously unhydrated cement particles and secondary reactions involving residual silica fume (SF) particles and calcium hydroxide, forming C-S-H-like phases^47,58,59^. The greater strength gains in furnace-cooled specimens may be attributed to the slower cooling process, which sustains the thermal conditions conducive to rehydration reactions, whereas rapid cooling in water may hinder these beneficial effects. The highest compressive strength recorded at this temperature was 89 MPa, achieved after 30 min of furnace soaking. This strength enhancement is a manifestation of competing physicochemical processes. At 200 °C, the dominant mechanism is the thermal activation of the system. The heat energy provides the necessary activation for further hydration of previously unhydrated cement grains (clinker phases like C₃S and C₂S) and accelerates the pozzolanic reaction between silica fume (SF) and portlandite (Ca(OH)₂). This leads to the deposition of additional C-S-H gel, which densifies the microstructure and enhances strength. The slower cooling rate in the furnace allows these beneficial exothermic reactions to proceed for a longer duration and promotes a more uniform temperature distribution, minimizing thermal gradients that can induce microcracking. In contrast, water quenching rapidly halts these reactions and induces significant thermal shock. The sudden contraction of the hotter surface relative to the cooler core generates tensile stresses, creating a network of microcracks that slightly offsets the strength gains from thermal activation.

At 400 °C, as shown in Fig. 5, the furnace-cooled specimens demonstrated strength increases of 13.8%, 7.9%, and 1.9% for soaking times of 10, 20, and 30 min, respectively, compared to room temperature values. These findings reveal a decreasing trend in strength gain with increasing soaking time. The peak compressive strength at 400 °C was 86.5 MPa, observed at a 10-minute soaking duration under furnace cooling. The enhancement in compressive strength observed at 400 °C can be attributed to either increased stiffness within the cementitious gel structure or intensified surface forces among gel particles due to the release of adsorbed water^24,25^. According to Djaknoun et al.^33^, high-strength mortar specimens subjected to heating at a rate of 3.3 °C/min for two hours and subsequently air-cooled exhibited an increase in mechanical strength up to 300 °C, after which a decline was observed at higher temperatures. Several studies have linked this improvement to continued hydrothermal reactions of unhydrated cement grains and the pozzolanic activity of silica fume (SF) with calcium hydroxide^51,60–63^. The temperature range at which compressive strength begins to decline varies and is influenced by multiple factors, including the binder-to-aggregate ratio, heating rate, water-to-binder ratio, and specimen size and shape^24^. At 400 °C, the results demonstrated that extending the exposure duration led to a progressive decrease in residual compressive strength, consistent with findings reported by Culfik and Ozturan^32^. This decline is likely associated with an increase in average pore size caused by the breakdown of hydration products, which leads to pore coarsening and subsequently reduced strength^31,64,65^. Recent investigations^66,67^ have further confirmed the inverse relationship between pore volume and compressive strength in hardened cement paste. Additionally, the degradation of concrete’s mechanical properties may occur without extensive material decomposition, driven instead by elevated temperatures, internal vapor pressure, and thermally induced stresses^68–70^. The transition from strength gain to loss with increasing soaking time at 400 °C is a critical indicator of shifting dominant mechanisms. The initial strength gain at short durations (10 min) is likely due to the same thermal activation process as at 200 °C, but now superimposed on the onset of detrimental processes. The key change is the initiation of dehydration reactions. Portlandite (Ca(OH)₂) begins to decompose (Ca(OH)₂ → CaO + H₂O) above ~ 400 °C, and the more tightly bound water in the C-S-H gel starts to be driven off. Initially, the loss of water may lead to a temporary stiffening of the C-S-H framework. However, with prolonged exposure (20–30 min), the detrimental effects dominate: the decomposition of hydration products creates a more porous and coarsened microstructure. The escape of water vapor creates internal pressure, enlarging existing pores and forming new microcracks. The longer the specimen is held at this elevated temperature, the more extensive this microstructural degradation becomes, explaining the progressive decline in strength with increased soaking time. The furnace cooling method, by maintaining the specimen at a high temperature for an extended period during slow cooling, effectively increases the total thermal dose, thereby exacerbating this degradation compared to a scenario where the damaging temperature is quickly reduced. Residual compressive strength measurements could not be obtained for C0 specimens cooled in water at 400 °C, nor for specimens exposed to 600 °C under either water or furnace cooling, due to explosive spalling that occurred under these conditions.

**Table 3:** Average compressive strength of C0 and C50 mixtures at room temperature and following furnace cooling.

Mixture	Compressive strength after furnace cooling (MPa)											
	RT		200 °C			400 °C			600°C			
	7	28	10	20	30	10	20	30	10	20	30	
**C-0**	61	76	81	85.5	89	86.5	82	77.5	-	-	-	
**C-50**	26.5	31.5	32.9	33.5	35	29.8	28.2	26.5	-	-	-	

### Influence of soaking duration and cooling method on the compressive strength of the C50 mixture

**Fig.6:**
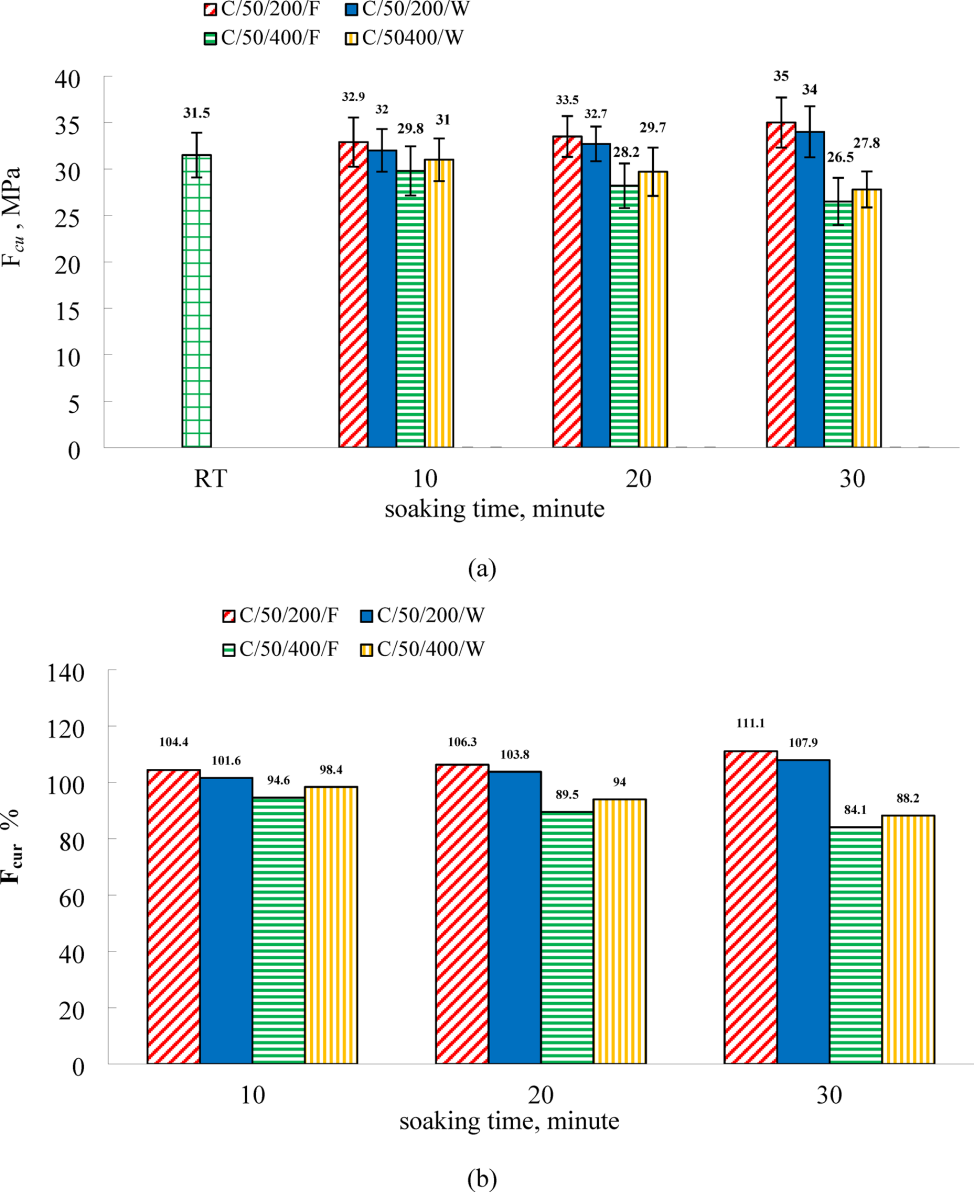
(**a**) The residual compressive strength for C50, (**b**) relative residual compressive strength for C50.

Figure 6 presents the residual compressive strength trends of C50 specimens subjected to furnace and water cooling. At ambient conditions, the compressive strength values for the C50 mix were 26.5 MPa at 7 days and 31.5 MPa at 28 days, as shown in Table 1.. In comparison to the C0 mix, the use of EPS as a lightweight aggregate in the C50 mixture led to reductions in compressive strength of 56.5% and 58.5% at 7 and 28 days, respectively. This reduction is primarily due to the minimal mechanical contribution and high compressibility of EPS particles^12,71^. At 200 °C, the compressive strength increased relative to room temperature for both cooling regimes. For furnace-cooled specimens, the increases were 4.4%, 6.3%, and 11.1% at soaking durations of 10, 20, and 30 min, respectively. In contrast, water-cooled specimens showed smaller gains of 1.6%, 3.8%, and 7.9% for the same soaking times. These findings indicate that compressive strength improves with longer exposure durations and that furnace cooling results in greater strength retention than water cooling. Both C0 and C50 mixtures followed a similar trend of strength enhancement at 200 °C under both cooling methods. However, the magnitude of increase was consistently higher in C0 specimens, as reported in Table 1.. This discrepancy can be attributed to the insulating nature of EPS, The attenuated strength gain in C50 at 200 °C is a direct consequence of its composite nature. The EPS beads, with their extremely low thermal conductivity, act as internal barriers to heat flow. This retards the rate at which the cementitious matrix between the beads reaches the temperatures required for optimal thermal activation and continued hydration. Consequently, the beneficial formation of additional C-S-H is less extensive than in the homogeneous, highly conductive C0 matrix. The mechanism of strength gain is the same, but its *efficacy* is reduced due to the physical obstruction and thermal insulation provided by the EPS, which limits heat transmission into the matrix and consequently reduces the extent of beneficial thermal effects, such as the rehydration of unreacted cement and silica fume that contribute to C–S–H formation. At this temperature, the highest compressive strength recorded for C50 was 35 MPa after 30 min of furnace exposure.

At 400 °C, a reduction in compressive strength was observed for C50 specimens under both cooling conditions. For furnace-cooled specimens, the strength decreased by 5.4%, 10.5%, and 15.5% at soaking durations of 10, 20, and 30 min, respectively. Water-cooled specimens showed smaller reductions of 1.6%, 6.0%, and 11.8% for the corresponding soaking times. These results demonstrate that increasing the soaking duration leads to a progressive decline in residual compressive strength, with the decline being more pronounced under furnace cooling than water cooling. Figure 6 highlights the impact of EPS on compressive strength, showing consistent reductions at all exposure durations and for both cooling methods, a trend that differs from the behavior of the C0 specimens. This reduction is attributed to the thermal degradation of EPS within the matrix, which results in increased porosity and the formation of microcracks, weakening the structural integrity of the material. At 400 °C, the behavior of C50 is governed by the thermal degradation of the EPS beads themselves. EPS undergoes pyrolysis and eventually vaporization at temperatures approaching 400 °C. This process leaves behind vacant spaces (voids) and, crucially, creates interconnected pathways of microcracks radiating from the sites of the decomposed beads. This drastically increases the total porosity and compromises the mechanical integrity of the composite. The reason water-cooled specimens show less strength loss than furnace-cooled ones is twofold. Firstly, the rapid quenching halts the EPS decomposition process earlier, limiting the total volume of EPS that is vaporized. Secondly, and more importantly, the water quenching promotes the rehydration of any free lime (CaO) formed from the decomposition of portlandite. The reaction CaO + H₂O → Ca(OH)₂ results in a volumetric expansion that can partially seal the newly formed microcracks and pores, leading to a temporary re-densification of the matrix and a better preservation of residual strength. Furnace cooling does not provide the moisture for this beneficial rehydration and allows for a slower, more complete degradation of the EPS, leading to greater strength loss. Furthermore, the insulating effect of remaining EPS limits the beneficial influence of heat, such as further hydration of cementitious materials. Lo Monte et al.^57^ reported that EPS-modified concrete tends to be more sensitive to elevated temperatures and generally exhibits lower mechanical strength at ambient conditions compared to conventional concrete. In agreement with the current results, Koskal et al.^50^ found that high-strength mortar specimens cooled in water after thermal exposure at 300 °C and 600 °C retained higher compressive strength than those cooled in air. This phenomenon is attributed to the decomposition of calcium hydroxide (Ca (OH)₂) into calcium oxide (CaO) at high temperatures, followed by its rehydration to Ca (OH)₂ during water cooling. The newly formed Ca (OH)₂ may fill microstructural voids and enhance matrix density, thereby improving residual strength^41,44–50^. In addition, prolonged thermal exposure causes a coarsening of the microstructure, contributing to strength degradation. Water cooling limits this effect by enabling faster temperature reduction, in contrast to furnace cooling, which extends the exposure period and accelerates microstructural damage^31,64,65^. It should be noted that residual compressive strength data for C50 specimens subjected to 600 °C could not be recorded under either cooling regime, as explosive spalling occurred at approximately 550 °C.

### Influence of soaking duration and cooling method on the splitting tensile strength of the C0 mixture

**Fig.7:**
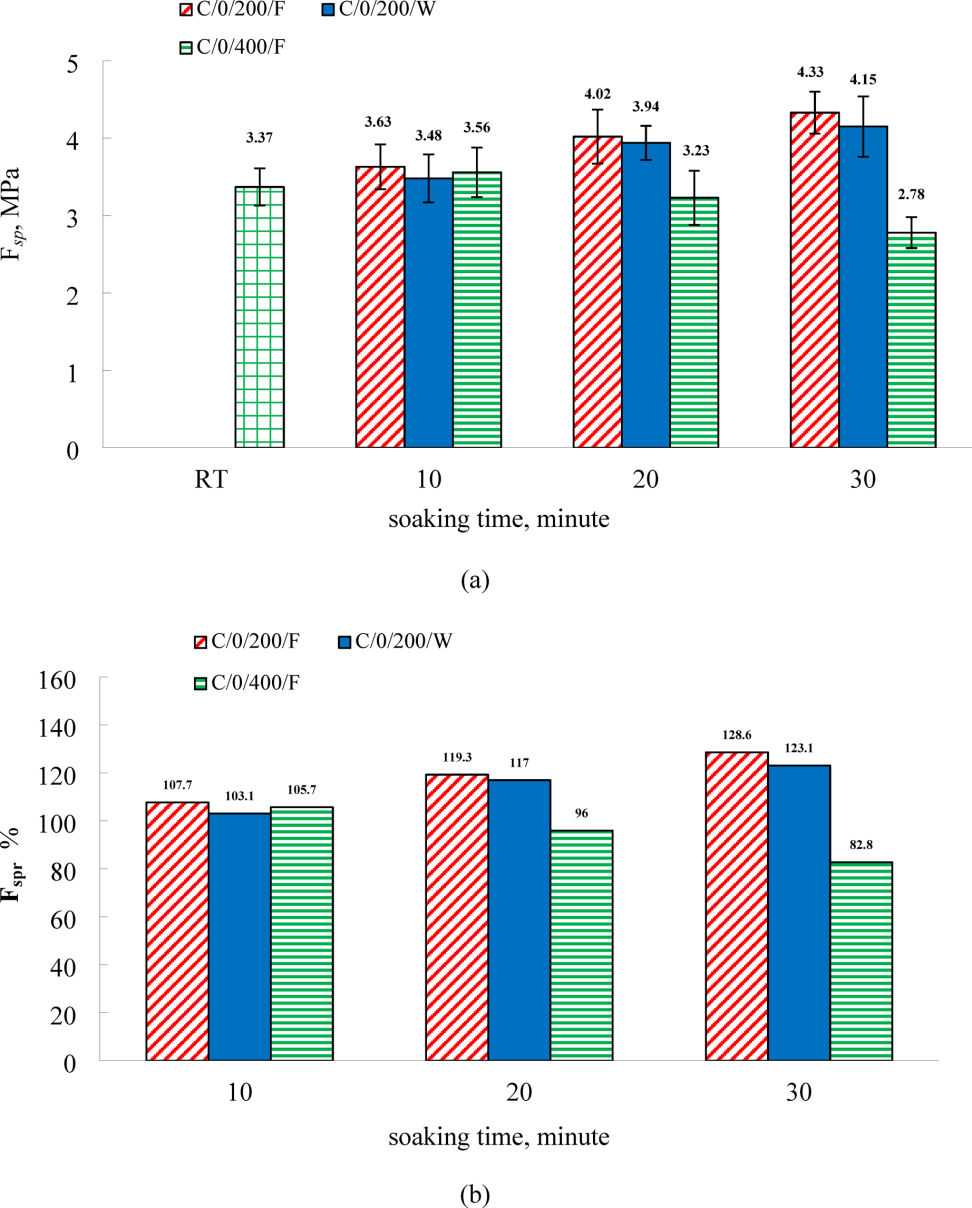
(**a**) The results of residual splitting tensile strength and (**b**) relative residual splitting tensile strength of C0 specimens.

Figure 7 illustrates the variation in residual splitting tensile strength of C0 specimens subjected to furnace and water cooling. As presented in Table 4., the splitting tensile strength at room temperature measured 2.56 MPa and 3.37 MPa at 7 and 28 days, respectively. At 200 °C, a strength increase was observed in furnace-cooled specimens, with improvements of 7.7%, 19.3%, and 28.6% recorded at soaking durations of 10, 20, and 30 min, respectively. Water-cooled specimens exhibited corresponding increases of 3.1%, 17.0%, and 23.1%. These results indicate that prolonged exposure time enhances the residual splitting tensile strength under both cooling regimes, with more significant gains observed in furnace-cooled specimens. This trend aligns with the behavior noted for compressive strength. In contrast to coarse aggregates, fine aggregates are relatively unaffected by elevated temperatures up to approximately 550 °C. Within this range, thermal and chemical alterations predominantly affect the cement paste. It is noteworthy that sand experiences a marked volumetric expansion at around 573 °C, associated with the phase transformation from alpha-quartz to beta-quartz, resulting in an approximate 0.85% volume increase. Therefore, below this transformation threshold, sand has a limited role in the deterioration of mechanical properties in high-strength mortar^72^.

The more severe degradation of splitting tensile strength compared to compressive strength at 400 °C is a fundamental reflection of the different mechanical properties they represent. Compressive strength is primarily influenced by the load-bearing capacity and friction between particles, which can be temporarily maintained even as microcracks begin to form. Tensile strength, however, is extremely sensitive to the presence of flaws and microcracks, as it directly measures the material’s resistance to crack propagation.

The decomposition of hydration products at 400 °C (e.g., ettringite, C-S-H dehydration, portlandite decomposition) creates a network of fine microcracks and increases porosity. While this network slightly reduces compressive capacity, it catastrophically impairs tensile capacity. Each microcrack acts as a stress concentrator, drastically reducing the force required to initiate and propagate a fatal crack under tensile loading. The longer the soaking time, the more extensive this microcrack network becomes, explaining the sharp decline in tensile strength with duration, even while compressive strength values may still be above the baseline. This highlights that tensile properties are a more sensitive indicator of early thermal damage in cementitious materials.

As illustrated in Fig. 7, exposure to 400 °C had a more pronounced adverse effect on the splitting tensile strength of C0 specimens compared to their compressive strength. While all furnace-cooled specimens exhibited compressive strength gains at this temperature across all soaking durations (10, 20, and 30 min), only those subjected to 10 min of heating showed a slight improvement in splitting tensile strength, with an increase of 5.7%. In contrast, specimens exposed for 20- and 30-minutes experienced reductions in residual tensile strength of 4% and 17%, respectively. This difference in behavior may be attributed to the greater sensitivity of splitting tensile strength to the development of microcracks and voids resulting from the decomposition of hydration products at elevated temperatures^24,73^. At 400 °C, the observed trend indicates that residual splitting tensile strength diminishes with increasing exposure time. Previous studies have similarly reported that the flexural strength of high-strength mortar, when air-cooled, improves up to 300 °C, followed by a gradual decline at higher temperatures^24^.

**Table 4:** Average splitting tensile strength of C0 and C50 mixtures at room temperature and following furnace cooling.

Mixture	Splitting tensile strength (MPa)										
	RT		200 °C			400 °C			600 °C		
	7	28	10	20	30	10	20	30	10	20	30
C-0	2.56	3.37	3.63	4.02	4.33	3.56	3.23	2.78	-	-	-
C-50	1.91	2.13	2.39	2.594	2.744	1.92	1.68	1.349	-	-	-

### Influence of soaking duration and cooling method on the splitting tensile strength of the C50 mixture

**Fig.8:**
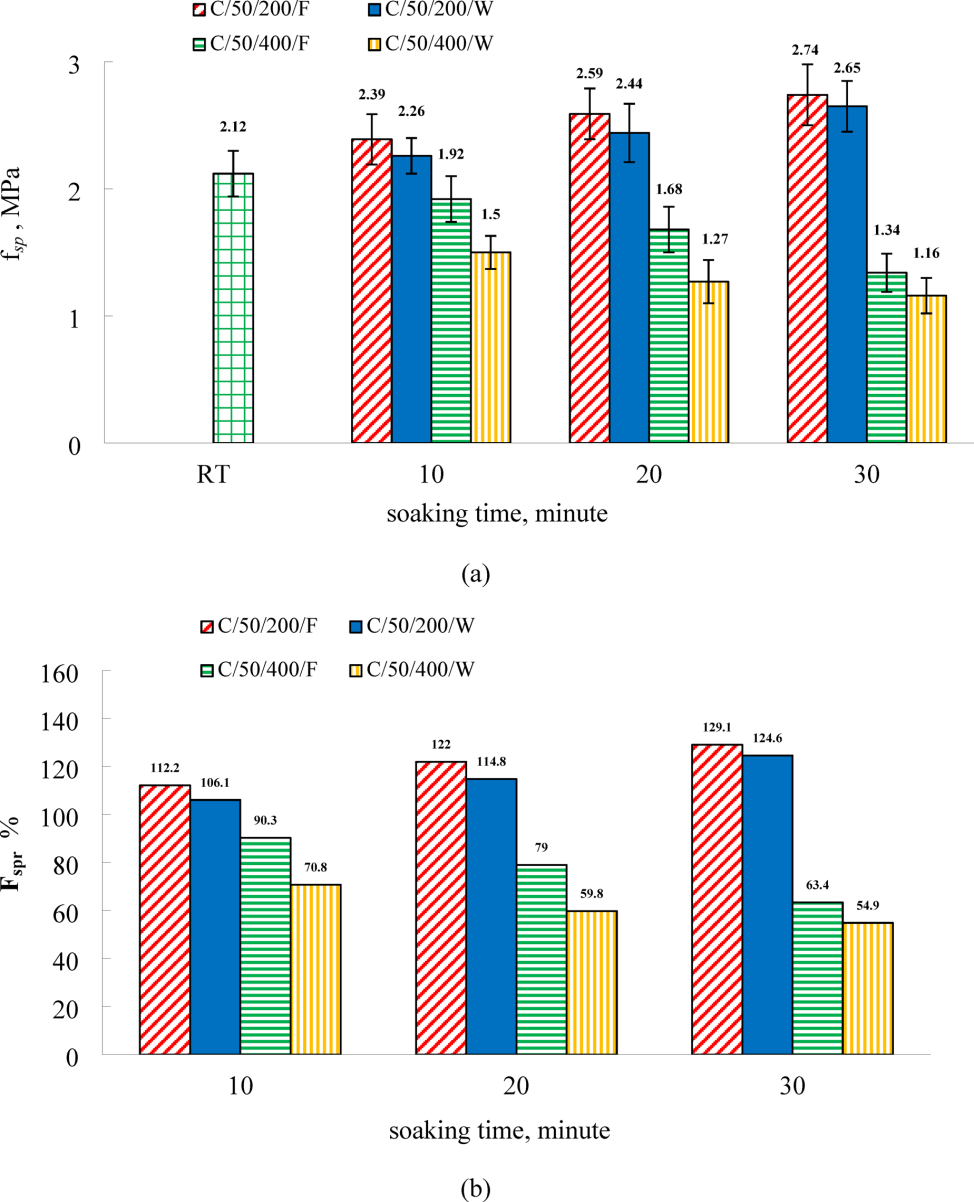
(**a**) The results of residual splitting tensile strength and (**b**) relative residual splitting tensile strength of C50 specimens.

Figure 8 illustrates the variation in residual splitting tensile strength of C50 specimens subjected to furnace and water cooling. As reported in Table 4, the splitting tensile strength at room temperature was 1.91 MPa and 2.13 MPa at 7 and 28 days, respectively. In comparison to the C0 mix, the inclusion of EPS as a lightweight aggregate in the C50 mixture led to a reduction in splitting tensile strength at ambient conditions by 25.4% and 36.8% at 7 and 28 days, respectively. At 200 °C, relative to room-temperature values, furnace-cooled specimens exhibited increases in splitting tensile strength of 12.2%, 22.0%, and 29.1% at soaking durations of 10, 20, and 30 min, respectively. Water-cooled specimens showed respective increases of 6.1%, 14.8%, and 24.6%. These results reflect a consistent trend observed in compressive strength measurements: residual tensile strength improves with prolonged exposure time, regardless of the cooling regime applied. Nonetheless, furnace-cooled specimens demonstrated a more pronounced enhancement in tensile strength compared to those cooled in water.

At 400 °C, the splitting tensile strength of C50 specimens decreased relative to room-temperature values. For furnace-cooled specimens, the reductions were 9.7%, 21.0%, and 36.6% at soaking times of 10, 20, and 30 min, respectively. In the case of water-cooled specimens, the corresponding decreases were more significant, reaching 29.2%, 40.2%, and 45.1%. These results confirm that prolonged thermal exposure leads to a progressive decline in residual splitting tensile strength, regardless of the cooling method employed. Unlike the compressive strength behavior observed at the same temperature, the splitting tensile strength suffered greater losses in water-cooled specimens compared to those cooled in the furnace. This difference is attributed to the thermal shock induced by sudden water immersion, which generates internal stresses and promotes the development of additional microcracks^43,51^. Koskal et al.^50^ similarly reported that water cooling results in a greater reduction in flexural strength than air cooling, due to the increased brittleness of mortar specimens subjected to abrupt temperature changes. Furthermore, the data indicate that the C50 mixture experienced a more substantial reduction in splitting tensile strength compared to the C0 mixture under both cooling conditions. This enhanced degradation can be linked to the increased porosity and microcracking within the matrix, resulting from the thermal decomposition of EPS particles during heating.

The catastrophic loss of tensile strength in C50, particularly under water cooling, is a synergistic failure of the composite matrix. The thermal decomposition of EPS beads creates large, inherent flaws (voids) and an interconnected network of microcracks. This severely weakens the matrix and provides easy paths for crack propagation. Under water quenching, a second, devastating mechanism is introduced: thermal shock. The sudden cooling causes the hot cementitious skeleton to contract rapidly. However, the vacant spaces left by the vaporized EPS cannot provide any restraint or support. This creates immense localized tensile stresses around these voids, forcing the pre-existing microcracks to widen and propagate instantly. The result is a brittle, fractured matrix with minimal residual tensile capacity.

In furnace cooling, the absence of this severe thermal shock avoids the instantaneous crack propagation. However, the slow cooling allows for more complete EPS degradation and extended exposure to high temperatures, leading to a more uniformly degraded and porous—though not as violently shattered—matrix, hence the slightly better but still poor performance compared to water quenching. This demonstrates that for EPS-composites in tension, the degradation mechanism (EPS decomposition) is so severe that the secondary mechanism (rehydration during water cooling) that benefits compressive strength is completely overwhelmed by the detrimental effects of thermal shock.

### Influence of soaking duration and cooling method on the impact energy of the C0 mixture

**Fig.9:**
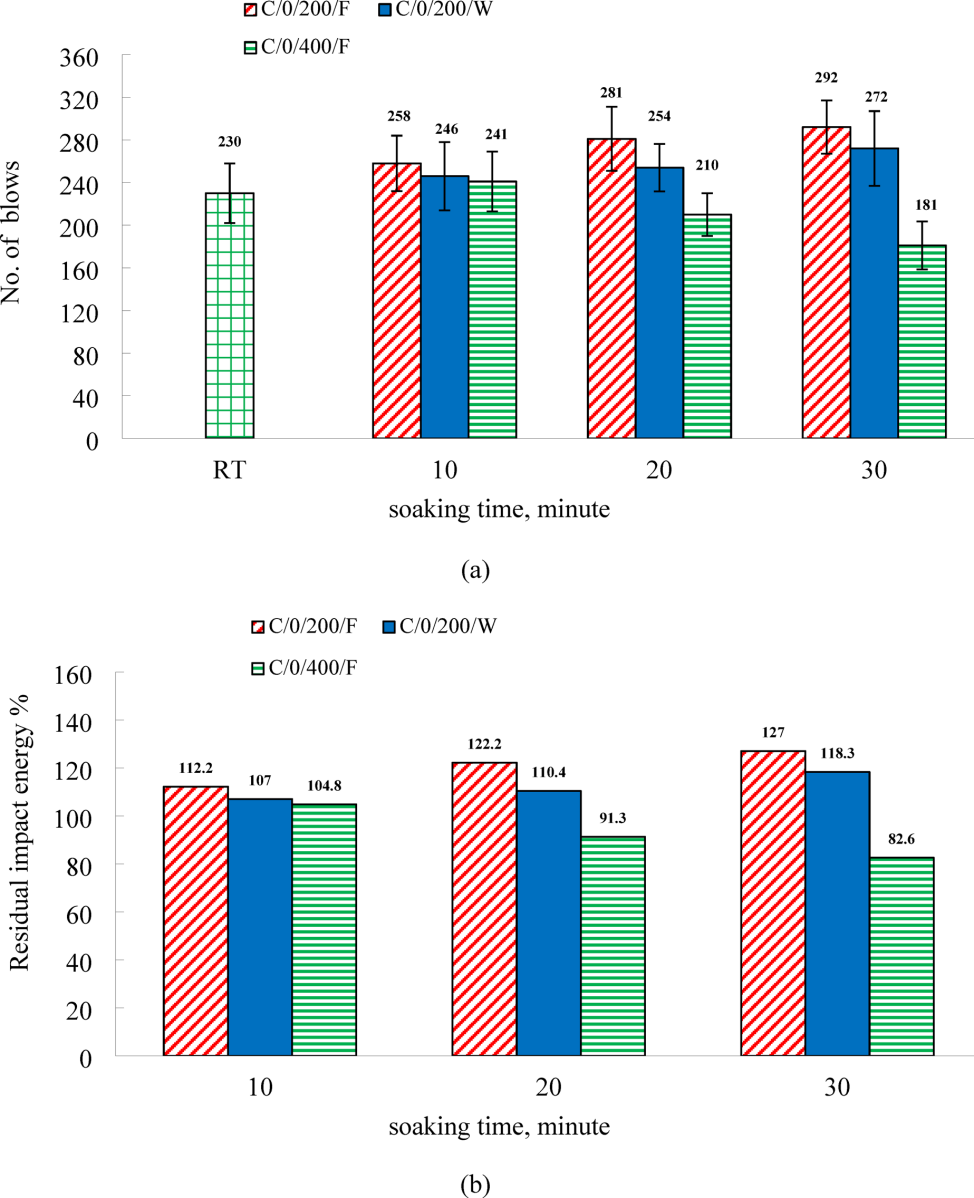
(**a**) The results of impact resistance and (**b**) relative residual impact energy of C0 specimens.

The drop weight impact test conducted on both C0 and C50 mixtures showed that all specimens exhibited only an initial visible crack (Ni), without progressing to a complete structural failure. This behavior is primarily linked to the inherent brittleness of the high-strength matrix, which limits its resistance to crack propagation. Moreover, the inclusion of EPS, characterized by its low mechanical resistance, did not impede crack growth. As a result, once the initial crack appeared, complete failure was immediate, leading to the assumption that Ni = Nf for calculating the impact energy. Figure 9 presents the residual impact energy of C0 specimens subjected to both furnace and water cooling for soaking durations of 10, 20, and 30 min. At room temperature, the impact energy values were 2764.88 kN·mm and 4675.9 kN·mm at 7 and 28 days, respectively, as shown in Table 5. At 200 °C, furnace-cooled specimens exhibited increases of 12.2%, 22.2%, and 27.0% in impact energy for the respective soaking durations, while the water-cooled counterparts showed increases of 7.0%, 10.4%, and 18.3%. These results indicate that longer thermal exposure enhances residual impact energy under both cooling conditions. However, specimens cooled in the furnace demonstrated superior improvements compared to those cooled in water, consistent with the strength development trends observed in compressive and tensile strength tests. As depicted in Fig. 9, exposure to 400 °C had a more pronounced negative impact on the residual impact energy of the specimens than on their compressive or splitting tensile strength. A slight increase of 4.8% in impact energy was observed only in the specimens subjected to 10 min of heating. In contrast, those exposed for 20 and 30 min exhibited reductions of 8.7% and 21.4%, respectively. These findings indicate that, at 400 °C, the residual impact energy declines progressively with increasing thermal exposure duration.

**Table 5:** Average impact energy values of C0 and C50 at room temperature and after furnace cooling.

Mixture	Impact energy (kN · mm)										
	RT		200 °C			400 °C			600 °C		
	7	28	10	20	30	10	20	30	10	20	30
C-0	2764.88	4675.9	5245.14	5712.73	5936.36	4899.53	4269.3	3679.73	-	-	-
C-50	731.88	1057.16	1158.81	1341.78	1382.44	650.56	569.24	325.28	-	-	-

**Table 6:** Average impact energy values of C0 and C50 mixtures following water cooling.

Mixture	Impact energy (kN · mm)								
	200 °C			400 °C			600 °C		
	10	20	30	10	20	30	10	20	30
C-0	5001.18	5163.82	5529.76	-	-	-	-	-	-
C-50	1097.82	1219.8	1280.79	528.58	365.94	203.3	-	-	-

### Influence of soaking duration and cooling method on the impact energy of C50 mixtures

**Fig.10:**
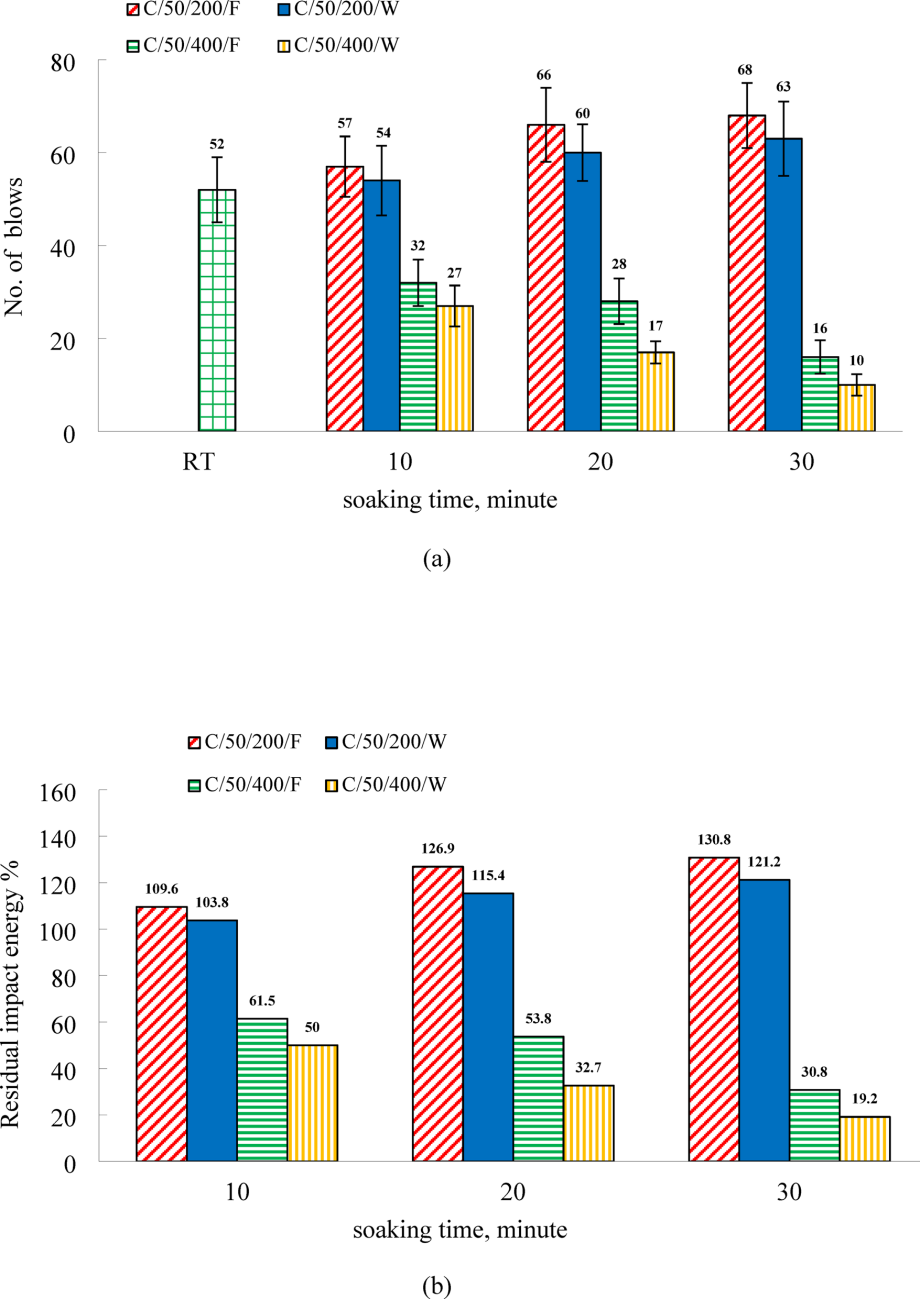
(**a**) The results of impact resistance and (**b**) the relative residual impact energy of C50 specimens.

Figure 10 illustrates the variation in impact energy of C50 specimens subjected to furnace and water cooling following soaking durations of 10, 20, and 30 min. At room temperature, the recorded impact energy was 731.88 kN·mm and 1057.16 kN·mm at 7 and 28 days, respectively, as indicated in Table 5. Compared to the C0 mixture, the incorporation of EPS as a lightweight aggregate in the C50 mix led to a reduction in room-temperature impact energy by 73.5% and 77.4% at 7 and 28 days, respectively. This reduction is attributed to the decreased compressive and tensile resistance of the C50 specimens^11^. Moreover, for complete failure to occur under impact loading, the applied energy must exceed the surface energy between opposing crack surfaces. EPS particles situated along the crack path act as weak zones due to their minimal mechanical strength, thereby lowering the surface energy required to propagate the crack. As a result, specimens containing EPS are more susceptible to failure under impact loads^11,74,75^. These findings are consistent with those of Zhao et al.^22^, who reported a significant decline in impact resistance when EPS content exceeded 40%. At 200 °C, impact energy for furnace-cooled specimens increased by 9.6%, 26.9%, and 30.8% for soaking times of 10, 20, and 30 min, respectively. Water-cooled specimens showed smaller gains of 3.8%, 15.4%, and 21.2% for the same durations. These results confirm that extended thermal exposure enhances residual impact energy, with furnace-cooled specimens exhibiting a more pronounced improvement. This trend mirrors the behavior observed in the residual compressive, tensile, and splitting tensile strengths. The improvement in impact energy at 200 °C, though modest, is a result of the material’s increased ability to absorb energy through micro-deformation rather than catastrophic cracking. The thermal activation process, which leads to further hydration and pore refinement, slightly toughens the brittle cementitious matrix. More significantly, the EPS beads, which remain largely intact at this temperature, play a crucial role. They act as compliant, energy-absorbing phases within the rigid matrix. Upon impact, these softer particles can deform elastically, absorbing and dissipating energy that would otherwise be concentrated at the tip of a propagating crack. This mechanism of stress redistribution and energy absorption enhances the overall impact resistance, turning the EPS from a mere flaw into a functional, energy-dissipating component at moderate temperatures.

At 400 °C, the impact resistance of furnace-cooled C50 specimens decreased by 38.5%, 46.2%, and 69.2% after soaking times of 10, 20, and 30 min, respectively. Water-cooled specimens exhibited even greater reductions of 50%, 67.3%, and 80.8% for the same durations. These results confirm that extended thermal exposure leads to a progressive decline in residual impact resistance, regardless of the cooling method employed. Consistent with the behavior observed in splitting tensile strength at this temperature, water cooling induced more severe degradation than furnace cooling. Furthermore, the loss in impact resistance at 400 °C was substantially higher than that recorded at 200 °C. This disparity is primarily due to the complete decomposition of EPS beads on the specimen surfaces at 400 °C across all heating durations. In contrast, at 200 °C, some EPS particles remained intact, contributing to improved impact resistance. The presence of EPS is thought to hinder crack propagation by slowing the growth of microcracks and distributing stresses more evenly throughout the matrix. Additionally, the random dispersion of EPS particles may help limit strain accumulation in regions experiencing stiffness degradation^76^.

The catastrophic loss of impact energy at 400 °C is a direct and severe consequence of the fundamental change in the role of EPS from a energy-dissipater to a defect-generator. The thermal decomposition of EPS beads vaporizes them, leaving behind large, sharp-edged voids. These voids are not merely empty spaces; they act as pre-existing critical flaws that drastically reduce the fracture energy of the composite.

Under the high-strain-rate loading of an impact test, cracks initiate almost instantaneously from these voids with minimal energy input. The mechanism of failure shifts from one requiring energy to *create* and *propagate* a crack to one where cracks simply *coalesce* between a network of pre-existing defects. The “surface energy” required for failure is thus negligible.

The even more severe performance under water cooling is due to thermal shock super-imposed on this already compromised microstructure. The rapid contraction during quenching generates intense tensile stresses that forcibly connect the EPS-derived voids and microcracks, creating a continuous fracture path through the specimen with virtually no resistance. This explains why the impact energy, a measure of total energy absorption, plummets to near-zero levels, indicating a complete loss of material cohesion and toughness.

### Weight loss

**Fig.11:**
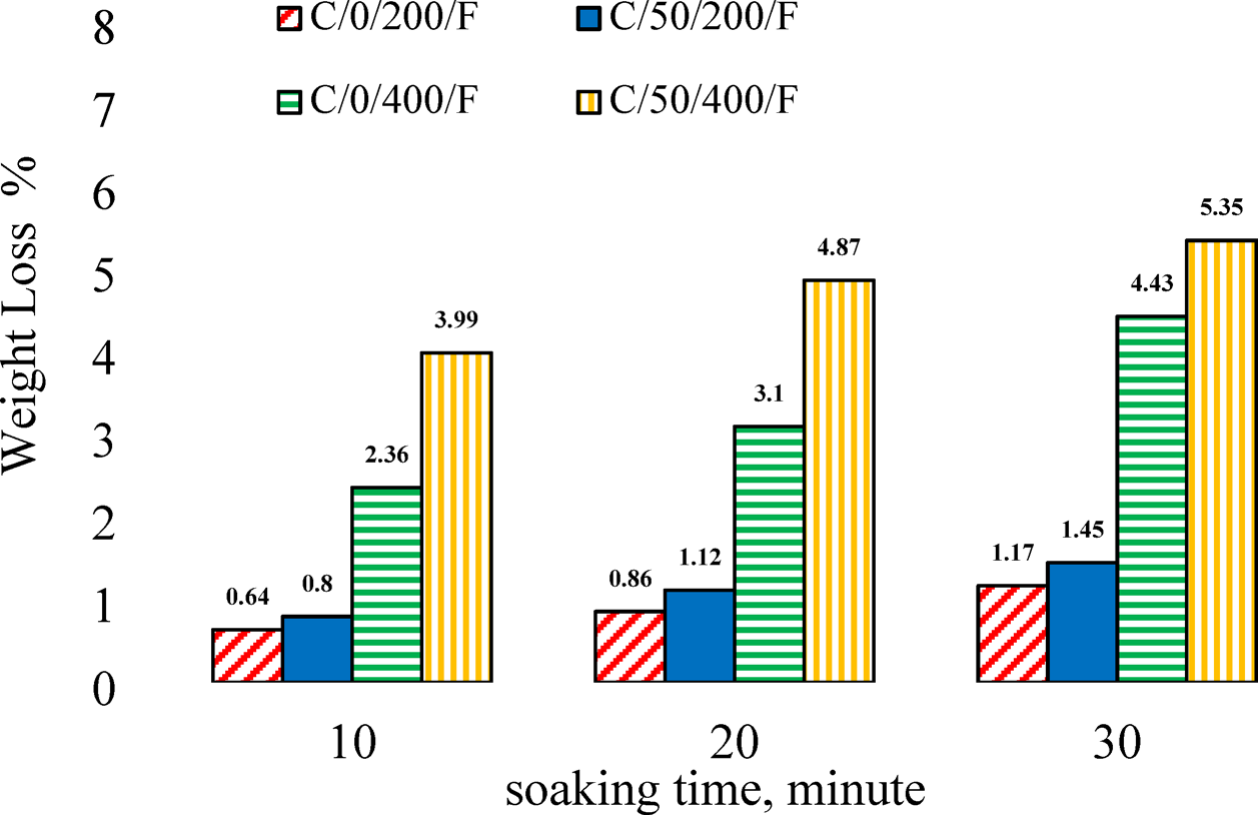
Mass loss of cubic specimens of C0 and C50.

Figure 11 illustrates the mass loss resulting from the evaporation of physically and chemically bound water in the dry unit weight of C0 and C50 specimens subjected to elevated temperatures and cooled in the furnace. Physically bound water begins to evaporate around 150 °C^77^, while the release of chemically bound water typically occurs at higher temperatures, often above 150–300 °C, depending on the cement composition^77^. Consequently, only slight changes in mass were observed at 200 °C, while more significant weight reductions were recorded at 400 °C and 600 °C. The results indicate a general decline in the density of both C0 and C50 specimens with increasing temperature. Additionally, prolonged exposure time led to further decreases in density, as shown in Fig. 11. The C50 specimens exhibited more pronounced mass loss compared to the C0 specimens, attributed to the thermal degradation of EPS granules, which contributed to increased porosity and microcrack formation, facilitating the escape of both types of bound water during heating. At room temperature, the average dry unit weights of the C0 and C50 specimens were approximately 2290 kg/m³ and 1755 kg/m³, respectively. Exposure of C0 specimens to 200 °C resulted in mass losses of 0.64%, 0.86%, and 1.17% at soaking times of 10, 20, and 30 min, respectively. Corresponding reductions for the C50 specimens were 0.8%, 1.12%, and 1.45%. At 400 °C, weight losses for C0 specimens reached 2.36%, 3.1%, and 4.43%, while C50 specimens exhibited higher losses of 3.99%, 4.87%, and 5.35% at the same respective soaking durations.

### The effect of EPS on thermal insulation properties

**Fig.12:**
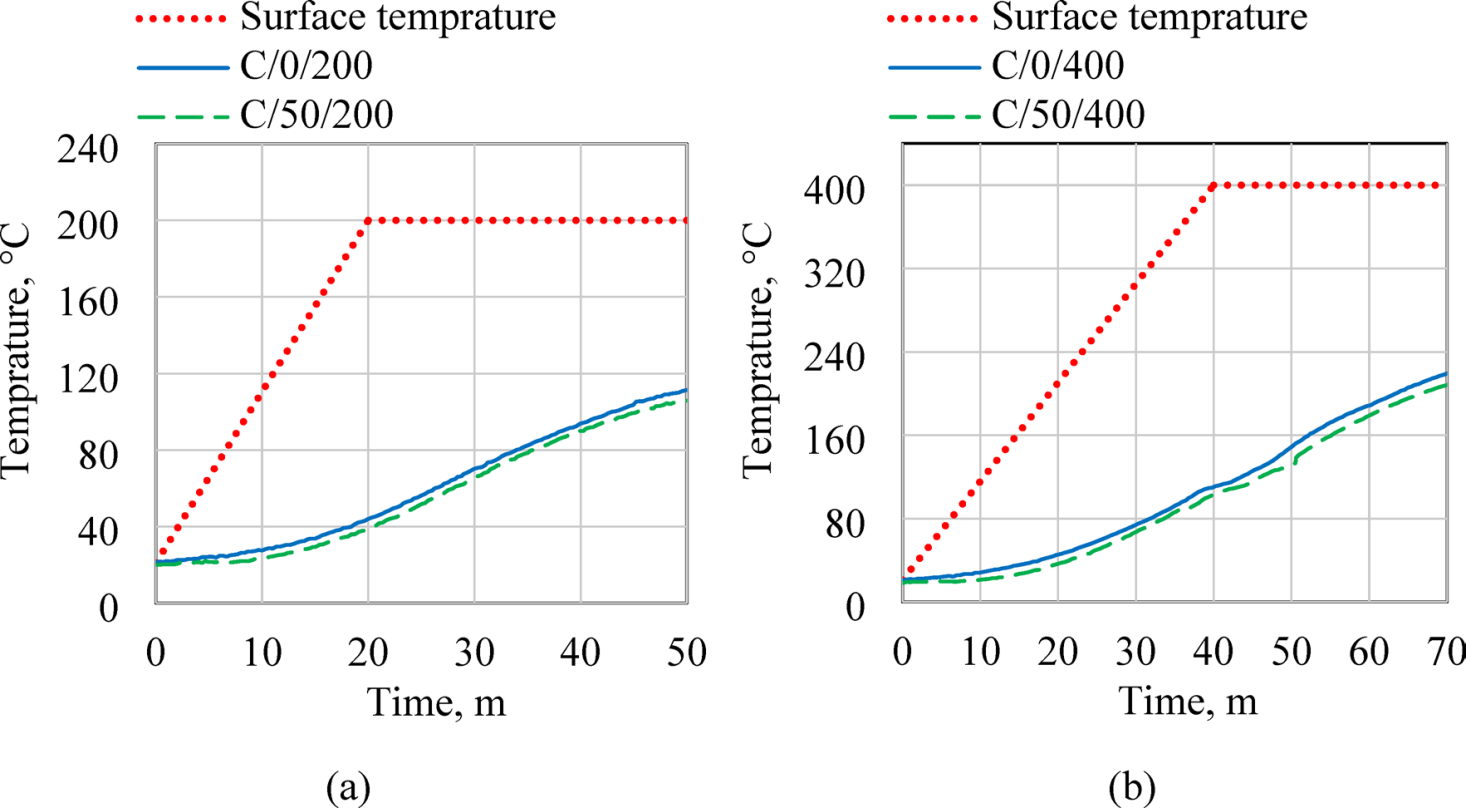
Temperature-time responses at the surface and core of C0 and C50 specimens under thermal exposure at (**a**) 200 °C and (**b**) 400 °C.

Figure 12 displays the impact of incorporating expanded polystyrene (EPS) as a lightweight aggregate on the thermal insulation performance of C0 and C50 specimens subjected to 30-minute exposure at 200 °C and 400 °C. The results demonstrate that the inclusion of EPS reduced the internal temperature rise, particularly within the first 15 min of heating, compared to the C0 mix. Additionally, the core temperatures recorded for C50 specimens were consistently lower than those of the corresponding C0 specimens, indicating enhanced insulation efficiency due to the presence of EPS. The reduction in thermal conductivity can be attributed to the inherent characteristics of EPS. As the effective thermal conductivity of insulation materials depends on the combined contributions of their solid and gas phases^78^, the incorporation of EPS—known for its low thermal conductivity—results in a noticeable decrease in overall heat transfer through the mortar. After 30 min of exposure, the core temperatures of the C50 specimens reached 106.25 °C and 208.5 °C when heated to 200 °C and 400 °C, respectively. Under the same conditions, the C0 specimens recorded higher core temperatures of 111.5 °C and 219.34 °C. The temperature gap between the two mixtures was 5.25 °C at 200 °C, increasing to 10.34 °C at 400 °C, further confirming the insulating effect provided by the EPS component.

### SEM analysis

**Fig.13:**
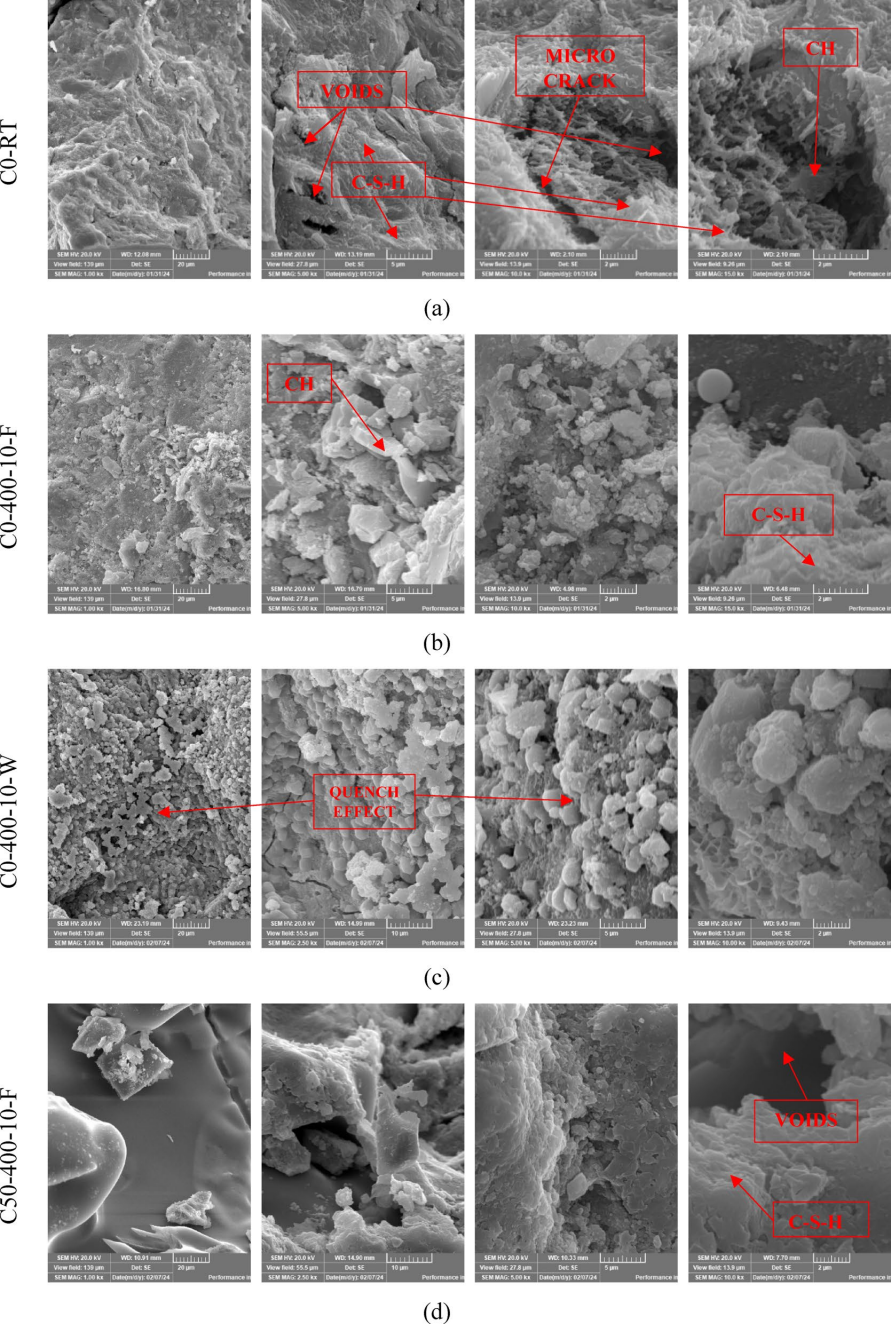

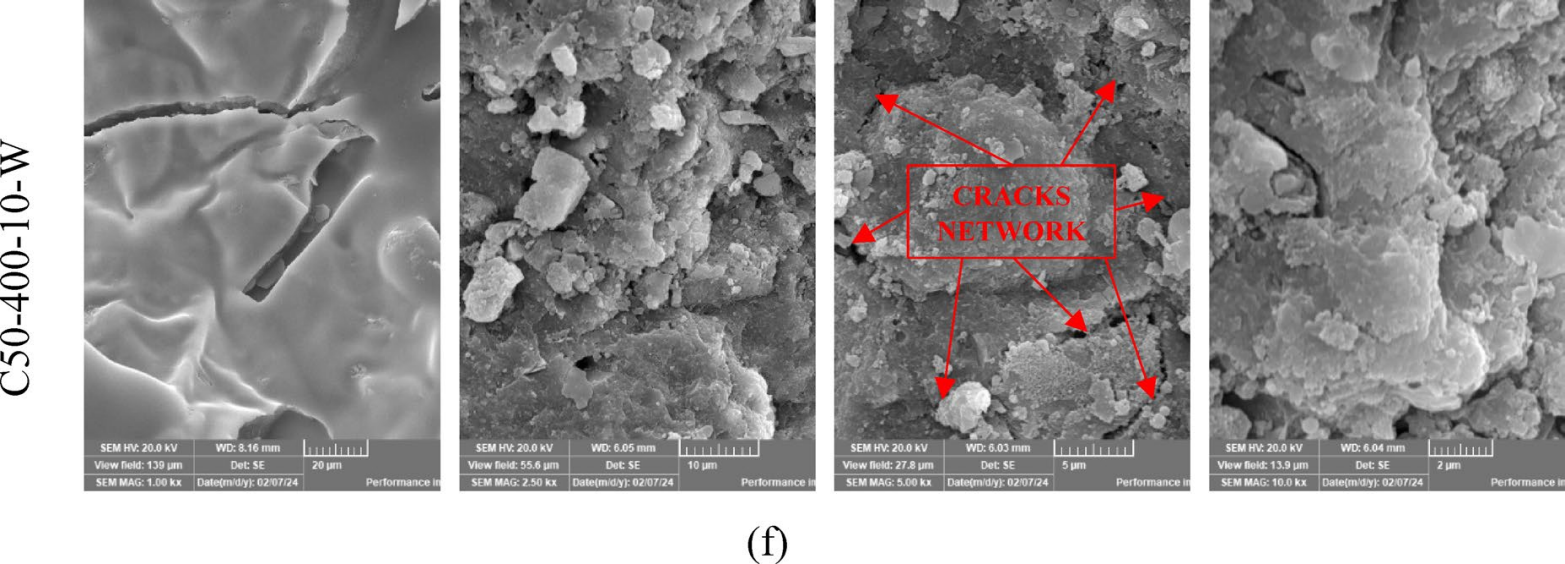
The SEM images of C0 and C50 specimens at different magnifications.

Scanning electron microscopy (SEM) analysis was performed on selected groups within this study to assess the microstructural changes in C0 and C50 mixtures subjected to elevated temperatures for short durations and different cooling methods. As depicted in Fig. 13, the analyzed samples include C0, C0-400-10-F, C0-400-10-W, C50-400-10-F, and C50-400-10-W. The SEM micrograph of the C0 specimen at ambient temperature (25 °C) reveals a microstructure dominated by microcrystalline and nearly amorphous calcium silicate hydrate (C–S–H). Furthermore, prominent calcium hydroxide crystals and dark regions corresponding to pores were identified. No visible microcracks were observed in the C0 specimen at 25 °C, as illustrated in Fig.13a.

As illustrated in Fig.13b, compared to the microstructure observed at room temperature, the specimens exposed to elevated temperatures (T = 400 °C) for 10 min and subsequently furnace-cooled exhibited a denser microstructure. This structural densification corresponded with a 13.8% increase in compressive strength, attributed to additional hydration facilitated by the evaporation of pore water during thermal exposure. Despite this strength gain, microcracks and voids were also identified, signaling the onset of microstructural coarsening. This may be associated with pore pressure buildup and the thermal degradation of tobermorite and calcium hydroxide phases^73^. Such coarsening and the increased porosity support the observed reduction in residual compressive strength with extended soaking durations^31^. As depicted in Fig.13c, the SEM image of the C0-400-10-W specimen (taken from an exploded sample) reveals more prominent microcracking compared to its furnace-cooled counterpart. These cracks are attributed to the thermal stresses induced by rapid cooling, which generated a significant temperature gradient between the specimen’s surface and core. Although rehydration of previously dehydrated phases led to a relatively compact microstructure, the pronounced microcracking resulting from water quenching significantly diminished the tensile strength compared to furnace-cooled specimens.

As depicted in Fig.13d and e, the SEM micrographs of the lightweight mixtures C50-400-10-F and C50-400-10-W, which incorporate EPS, reveal that the inclusion of EPS results in the development of additional voids, microcracks, and carbonaceous residues attributed to the thermal degradation of EPS particles. The cracks and voids surrounding the EPS beads indicate potential zones of weakness within the matrix that may adversely affect the mechanical integrity of the material. Furthermore, in comparison to C50-400-10-W, the SEM image of C50-400-10-F (Fig.13d) exhibits a higher concentration of voids, reflecting a more pronounced coarsening of the microstructure. This can be attributed to the extended cooling period associated with furnace cooling, which delays the rehydration of dehydrated phases. Conversely, the water-cooled specimens benefit from rapid temperature reduction, which enhances the rehydration process and promotes a denser microstructure. This distinction in microstructural development aligns with the observed improvement in compressive strength for water-cooled specimens relative to those cooled in the furnace.

The SEM analysis provides direct visual evidence of the physicochemical mechanisms governing performance. For C0-400-10-F (Fig. 13b), the denser microstructure confirms the occurrence of thermal activation and sintering, explaining the initial strength gain. However, the simultaneous presence of microcracks reveals the inception of detrimental processes like dehydration and internal pore pressure build-up, foreshadowing the strength loss observed with longer soaking times.

For C0-400-10-W (Fig. 13c), the extensive microcracking is the physical manifestation of thermal shock. These quench-induced cracks provide a clear mechanistic explanation for the severe loss in tensile strength, as they create direct pathways for failure under tension-dominated stresses.

For the C50 specimens (Fig. 13d, e), the micrographs unequivocally show the primary failure mechanism: the formation of massive, interconnected voids from vaporized EPS. The carbonaceous residues signify incomplete combustion, confirming the EPS degradation pathway. The cracks radiating from these voids show how they become the epicenters of failure. The key difference between furnace and water cooling is visible: C50-400-10-F shows more extensive void coarsening due to longer exposure to peak temperature, while C50-400-10-W shows a finer crack network emanating from the voids, evidence of the violent thermal shock that shattered the already weakened matrix.

**Fig.14:**
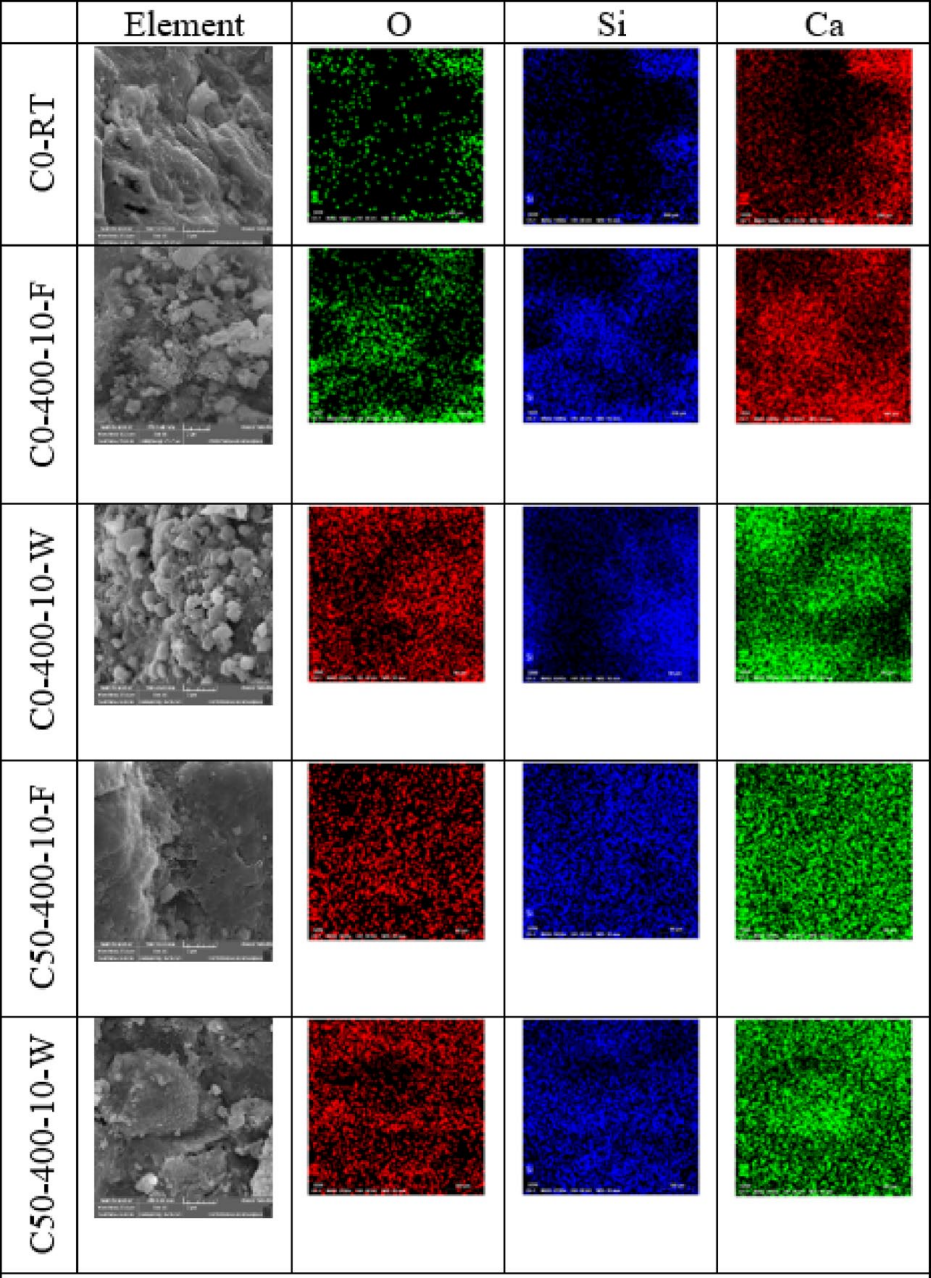
The elemental mapping of C0 and C50 specimens.

Energy Dispersive Spectroscopy (EDS) mapping was utilized to perform a detailed elemental analysis of the C0 and C50 matrices. As shown in Fig. 14, the overall elemental composition (primarily Ca, Si, O) at both ambient and elevated temperatures showed no significant gross migration or change. However, it is crucial to note that this macroscopic elemental stability does not imply microstructural or chemical phase stability. The observed mechanical degradation (Sect. 3.2–3.7) is driven by physicochemical processes such as the dehydration of C-S-H, decomposition of portlandite and ettringite, and thermal degradation of EPS, which alter the pore structure and cohesion of the matrix without necessarily changing its bulk elemental composition.

The EDS results reveal a critical insight: the bulk chemical stability masks profound phase changes. The decrease in Ca/Si ratio in C0 specimens, especially after water cooling, is a strong indicator of portlandite (Ca(OH)₂) consumption. This occurs through two simultaneous processes: (1) its decomposition into lime (CaO) at high temperature, and (2) the enhanced pozzolanic reaction with silica fume, both of which reduce the calcium content relative to silicon. The more pronounced effect in water-cooled samples suggests that quenching may accelerate the pozzolanic reaction by providing moisture, leading to a denser, more polymerized C-S-H gel with a lower Ca/Si ratio, which correlates with higher strength.

For C50 specimens, this narrative is completely overturned by physical degradation. While the chemistry (lowered Ca/Si ratio) suggests a *stronger* matrix, the physical reality—massive voids from EPS decomposition—completely dominates the mechanical performance. This creates a clear dichotomy: the chemical metrics indicate improvement, but the physical metrics (porosity, flaw size) indicate catastrophic degradation. This explains the paradox of why C50 strength plummets despite a seemingly favorable chemical evolution; the physical damage inflicted by EPS vaporization is the overriding factor controlling mechanical behavior. The EPS composite fails not because of chemical instability, but because of a catastrophic loss of physical integrity.

As shown in Table 7, energy-dispersive X-ray spectroscopy (EDS) was employed to measure the Ca/Si atomic ratio in C0 and C50 samples, providing insights into the chemical composition of C-S-H within the hydrated cement paste matrix. The formation and reactivity of Ca and Si ions during hydration play a crucial role in determining the structural evolution of C-S-H. A lower Ca/Si ratio correlates with a denser and more compact cement microstructure, enhancing the mechanical integrity of the C-S-H network. When the temperature increased from room temperature (RT) to 400 °C, the Ca/Si ratio for C0-400-10-F and C0-400-10-W slightly decreased compared to C0-RT, corresponding to a marginal increase in compressive strength at 400 °C. Notably, C0-400-10-W exhibited a more pronounced reduction in the Ca/Si ratio than C0-400-10-F, supporting the observation that water-cooled specimens achieved higher compressive strength than furnace-cooled ones. In contrast, while C50 samples at 400 °C (both C50-400-10-F and C50-400-10-W) displayed a reduced Ca/Si ratio compared to C0-RT—suggesting intrinsic matrix strengthening—their compressive strength declined across all exposure durations (10, 20, and 30 min) relative to RT values. This reduction is attributed to the thermal decomposition of expanded polystyrene (EPS) at elevated temperatures, which induces void formation and microcrack development. The results demonstrate that EPS undergoes rapid degradation at 400 °C, destabilizing hydration products such as calcium hydroxide and ettringite. This thermal instability adversely affects the composite’s mechanical performance, highlighting the critical influence of temperature on cement-based materials. These findings underscore the necessity for additional research into the durability and structural behavior of EPS-incorporated composites, particularly in high-temperature environments, to ensure their long-term reliability in practical applications.

It is noted that the use of fractured surfaces for EDS analysis, rather than polished cross-sections, may result in an overrepresentation of certain phases exposed by the fracture path. Therefore, these ratios should be interpreted as indicative of local chemical environments rather than the precise bulk chemistry of the C-S-H gel.

**Table 7:** The average ratios of Ca/Si in the matrix of C0 and C50 specimens.

ID	Ca/Si
C0-RT	3.33
C0-400-10-F	2.43
C0-400-10-W	1.26
C50-400-10-F	2.53
C50-400-10-W	1.75

## Conclusions

This study investigated the performance of high-strength and EPS-modified mortars under short-term, high-temperature exposure. The key findings, drawn from mechanical testing, thermal analysis, and microstructural evaluation, lead to the following conclusions:

1. Spalling Resistance: The incorporation of EPS beads effectively eliminated explosive spalling at 400 °C under all cooling regimes. The mechanism is attributed to the generation of additional porosity from partial EPS degradation, which provides pathways to dissipate internal vapor pressure. In stark contrast, the conventional high-strength mortar experienced catastrophic spalling.
2. Strength Performance: A fundamental temperature-dependent shift in performance was observed. At 200 °C, both mortar types exhibited strength gains due to thermal activation, though the gains were more modest in the EPS-modified mortar due to its insulating nature. At 400 °C, the EPS-mortar suffered progressive strength loss with increased exposure time, a direct result of matrix damage from EPS decomposition.
3. Thermal Insulation: The inclusion of EPS provided significant thermal insulation, substantially reducing the core temperature rise during heating. However, this benefit is counterbalanced by the associated reduction in mechanical strength and the physical degradation of the matrix at high temperatures.
4. Microstructural Evolution: Microanalysis confirmed that the EPS-induced voids are the primary factor governing behavior—beneficial for pressure relief at moderate temperatures but becoming critical defects that exacerbate degradation at extreme temperatures. Elemental analysis confirmed that the mechanical degradation was primarily a result of this physical breakdown rather than chemical compositional changes.
5. Practical Implications: EPS presents a viable strategy for mitigating explosive spalling in scenarios where temperatures are unlikely to exceed 400 °C. However, its application in high-temperature environments (≥ 550 °C) is not recommended without complementary protection measures, as its rapid decomposition can severely compromise structural integrity.
6. Limitations and Future Research: This study was limited to a single EPS content and size. Future work should investigate the effect of EPS bead size distribution, lower volume fractions, and the use of polystyrene fibers on spalling mitigation and residual properties under similar transient thermal conditions.

## Data Availability

All data generated or analyzed during this study are included in this published article.
